# CT radiomic signature predicts survival and chemotherapy benefit in stage I and II HPV-associated oropharyngeal carcinoma

**DOI:** 10.1038/s41698-023-00404-w

**Published:** 2023-06-02

**Authors:** Bolin Song, Kailin Yang, Vidya Sankar Viswanathan, Xiangxue Wang, Jonathan Lee, Sarah Stock, Pingfu Fu, Cheng Lu, Shlomo Koyfman, James S. Lewis, Anant Madabhushi

**Affiliations:** 1 Center for Computational Imaging and Personalized Diagnostics, Emory University, Atlanta, GA USA; 2 Department of Radiation Oncology, Taussig Cancer Center, Cleveland Clinic, Cleveland, OH USA; 3 School of Automation, Nanjing University of Information Science and Technology, Nanjing, China; 4 Imaging Institute, Cleveland Clinic, Cleveland, OH USA; 5 Department of Population and Quantitative Health Sciences, Case Western Reserve University, Cleveland, OH USA; 6 Department of Pathology, Microbiology, and Immunology, Vanderbilt University Medical Center, Nashville, TN USA; 7 Louis Stokes Cleveland Veterans Administration Medical Center, Cleveland, OH USA

**Keywords:** Predictive markers, Oral cancer detection, Mathematics and computing, Oral cancer

## Abstract

Chemoradiation is a common therapeutic regimen for human papillomavirus (HPV)-associated oropharyngeal squamous cell carcinoma (OPSCC). However, not all patients benefit from chemotherapy, especially patients with low-risk characteristics. We aim to develop and validate a prognostic and predictive radiomic image signature (pRiS) to inform survival and chemotherapy benefit using computed tomography (CT) scans from 491 stage I and II HPV-associated OPSCC, which were divided into three cohorts D_1_–D_3_. The prognostic performance of pRiS was evaluated on two test sets (D_2_, *n* = 162; D_3_, *n* = 269) using concordance index. Patients from D_2_ and D_3_ who received either radiotherapy alone or chemoradiation were used to validate pRiS as predictive of added benefit of chemotherapy. Seven features were selected to construct pRiS, which was found to be prognostic of overall survival (OS) on univariate analysis in D_2_ (hazard ratio [HR] = 2.14, 95% confidence interval [CI], 1.1–4.16, *p* = 0.02) and D_3_ (HR = 2.74, 95% CI, 1.34–5.62, *p* = 0.006). Chemotherapy was associated with improved OS for high-pRiS patients in D_2_ (radiation vs chemoradiation, HR = 4.47, 95% CI, 1.73–11.6, *p* = 0.002) and D_3_ (radiation vs chemoradiation, HR = 2.99, 95% CI, 1.04–8.63, *p* = 0.04). In contrast, chemotherapy did not improve OS for low-pRiS patients, which indicates these patients did not derive additional benefit from chemotherapy and could be considered for treatment de-escalation. The proposed radiomic signature was prognostic of patient survival and informed benefit from chemotherapy for stage I and II HPV-associated OPSCC patients.

## Introduction

There has been a significant increase in the incidence of HPV-associated oropharyngeal squamous cell carcinoma (OPSCC) over the past several decades^1^. Based on data from 2015 to 2019, about 47,199 new HPV-associated cancers occurred in the United States each year, accounting for about 70% of cancers of the oropharynx^1^. It has been shown that patients with HPV-associated OPSCC demonstrate improved treatment response to chemoradiation and prognosis compared to patients with HPV independent OPSCC, whose tumors are much more often associated with alcohol and tobacco use^2^. A distinct tumor, node and metastasis (TNM) staging system for HPV-associated OPSCC patients was established in the 8th edition of the American Joint Commission on Cancer (AJCC) staging system to account for the unique clinical characteristics^3^. However, recent studies report that the differentiation of outcome between the AJCC 8th stage groups remains less than satisfactory^4,5^. New biomarkers to improve AJCC 8th staging in risk stratification within the HPV-associated OPSCC population is sorely needed.

Previous studies have shown that using concurrent chemoradiation as definitive treatment confers lower local and regional failure rates compared with radiotherapy alone for high risk OPSCC. However, the addition of chemotherapy can cause increased treatment-related toxicity, a particular concern for patients with HPV-associated OPSCC who are at low risk for disease recurrence^6–8^. Skillington et al. compared the outcomes of 195 p16-positive, surgically managed OPSCC patients and found that receiving chemotherapy did not provide additional disease-free survival benefit and was associated with worse overall survival, potentially due to the lack of benefit from the additional chemotherapy in low-risk patients^6^. Furthermore, toxicity from the chemotherapy was also observed^9–11^. Significant deteriorations in swallowing outcome occurred in those who had chemotherapy in addition to radiotherapy^9^. To reduce treatment toxicity without compromising survival outcomes for HPV-associated OPSCC patients, recent clinical studies have focused on identifying patients suitable for treatment de-escalation with lower dose radiotherapy and also for potentially avoiding the use of chemotherapy without compromising survival outcome^12,13^. This highlights the need for developing a predictive biomarker to distinguish which low-risk OPSCC patients may not benefit from chemotherapy and thus for whom such toxic treatments could be avoided, without compromising patient outcomes.

With the advent of computerized image analysis and an increased attention paid to machine learning, there is an opportunity for deep mining of computer-extracted image features to inform patients’ outcome and to guide treatment intensities^14–17^. Radiomics, which seeks to identify subtle image-based attributes related to tumor phenotypes^18,19^ and prognosis^20,21^ is useful in not only identifying the presence of disease on radiographic scans but also in helping identify features that related to disease outcome and treatment response. Although a few studies have shown that machine learning based prognostic classifiers can predict molecular subtype^18,19^ and survival^20,21^ in head and neck cancer, there has been no methodology available to evaluate the predictive utilities of these approaches for chemotherapy benefit among HPV-associated OPSCC patients. Those patients with low risk of disease recurrence (AJCC 8th edition stage I and II patients) who would not derive additional benefit from chemotherapy could therefore potentially avoid the toxicity induced by chemotherapy.

In this work, we interrogate a prognostic and predictive radiomic image signature (pRiS) that employs quantitative texture features derived from within and around the primary oropharyngeal tumor on treatment planning CT scans to predict survival and chemotherapy benefit. Using a total of 491 patients from 4 sites, treated either with radiotherapy + chemotherapy or radiotherapy alone, our goal is to investigate if pRiS is (a) prognostic of survival and (b) associated with chemotherapy benefit in AJCC stage I and stage II HPV-associated OPSCC patients.

## Results

### Clinical characteristics

Table 1 lists the detailed demographic and clinical characteristics of the patients in cohorts D_1_ (*n* = 60), D_2_ (*n* = 162), and D_3_ (*n* = 269). Of the total 491 patients included in the study, 409 (83.3%) were men, and the median (interquartile range) age was 59 (53.8–64.2) years. 223 patients (45.4%) had AJCC 8th edition stage I OPSCC, of which 128 (57.4%) patients received chemotherapy. 268 patients (54.6%) had AJCC 8th edition stage II OPSCC, of which 201 (75%) patients received chemotherapy. More details regarding treatment are provided in Supplementary Table 2. Demographic and clinical characteristics according to treatment groups are provide in Supplementary Table 3. The median values for OS in D_1_, D_2_ and D_3_ were 7.48, 7.76, and 5.16 years and the median values for DFS were 7.06, 7.63, and 5.13 years.

**Table 1:** Demographic characteristics of the patients.

Parameter	D_1_ (*n* = 60)	D_2_ (*n* = 162)	D_3_ (*n* = 269)	*p* Value
Age	60.70 ± 10.21	59.07 ± 8.04	58.71 ± 8.98	0.21
Gender				
Male	47 (78.3%)	131 (80.1%)	231 (85.9%)	0.22
Female	13 (21.7%)	31 (19.9%)	38 (14.1%)	
Tumor subsite				
Base of tongue	18 (30%)	67 (41.4%)	140 (52%)	
Tonsillar complex	37 (61.7%)	89 (54.9%)	120 (44.6%)	0.01
Posterior wall/soft palate	5 (8.3%)	6 (3.7%)	9 (3.4%)	
Smoking PY	18.17 ± 19.20	16.59 ± 20.92	10.12 ± 15.71	<0.0001
T-stage				
T1	17 (28.3%)	33 (20.4%)	81 (30.1%)	
T2	23 (38.3%)	72 (44.4%)	129 (48.0%)	0.02
T3	20 (33.4%)	57 (35.2%)	59 (21.9%)	
N-stage				
N0	19 (31.7%)	14 (8.6%)	37 (13.8%)	
N1	34 (56.7%)	108 (66.7%)	111 (41.3%)	<0.0001
N2	7 (11.6%)	40 (24.7%)	121 (44.9%)	
AJCC 8th Stage				
I	36 (60.0%)	83 (51.2%)	118 (43.9%)	0.09
II	24 (40.0%)	79 (48.8%)	151 (56.1%)	
Recurrence				
Local/regional/distant	10 (16.7%)	20 (12.3%)	26 (10%)	0.27
Non-recurrence	50 (83.3%)	142 (87.7%)	243 (90%)	

### pRiS for prognosis prediction

**Fig. 1:**
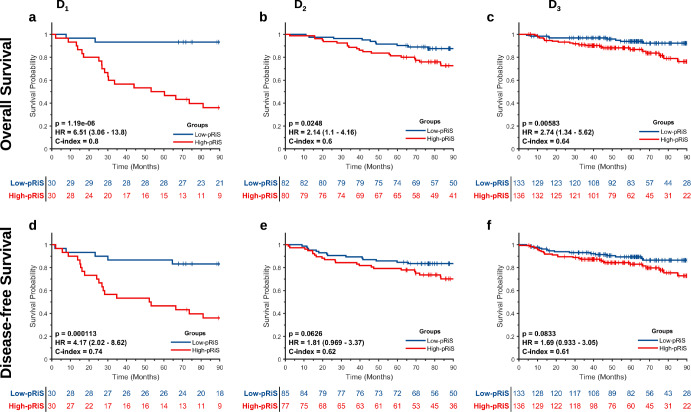
Kaplan–Meier survival analysis of pRiS on AJCC 8th stage I and II HPV-associated OPSCC. Stratifications are provided using overall survival from cohorts D_1_ (**a**), D_2_ (**b**), and D_3_ (**c**) and using disease-free survival from cohorts D_1_ (**d**), D_2_ (**e**), and D_3_ (**f**).

**Fig. 2:**
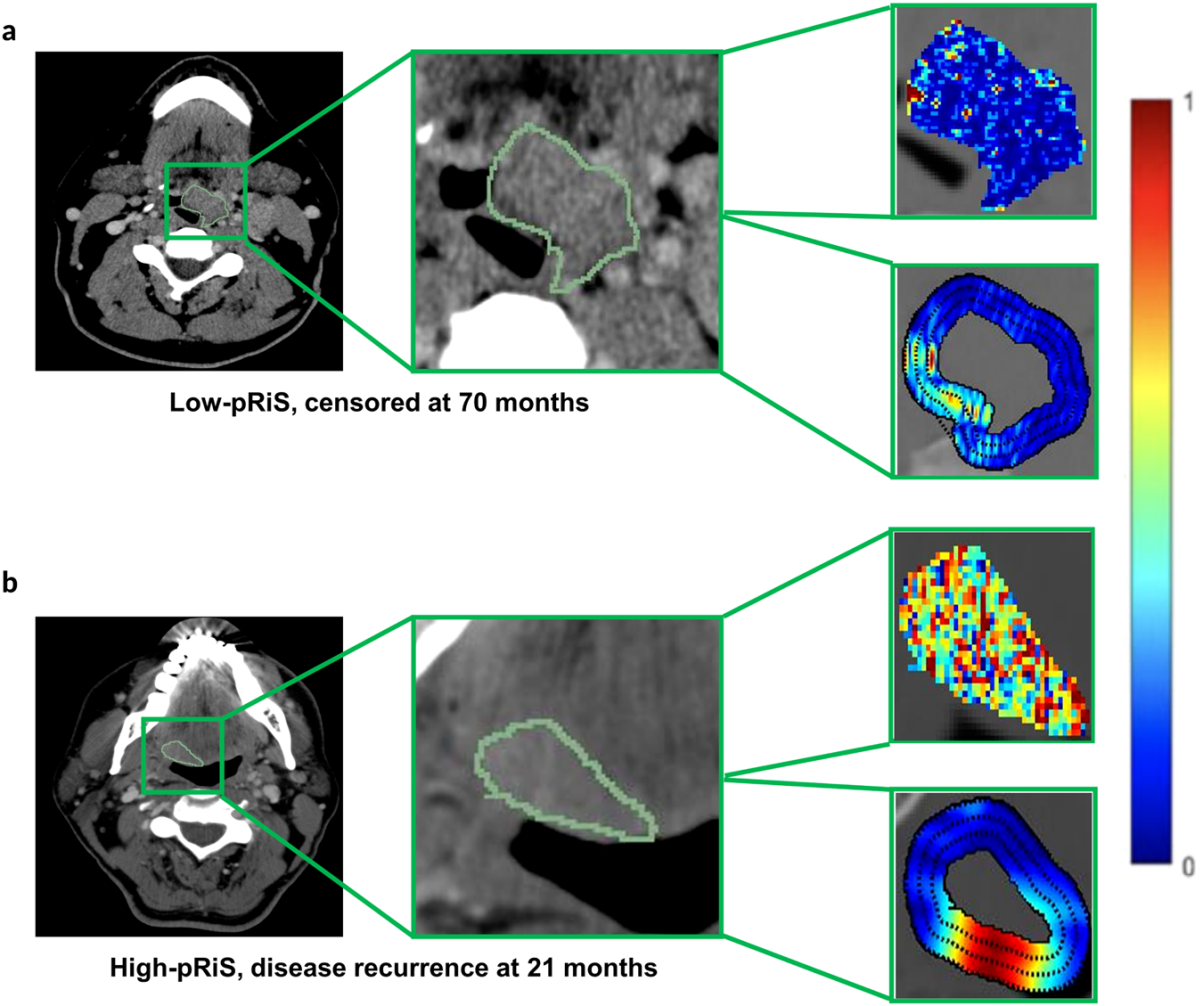
Intratumoral and peritumoral radiomic feature maps. Feature maps are overlaid onto the corresponding region of interests of an example low-pRiS patient (**a**) and an example high-pRiS patient (**b**). We observe stronger feature expressions in terms of Laws texture (intratumoral) and CoLlAGe inertia (peritumoral) in the high-pRiS patient compared to the low-pRiS patient.

Top selected features from LASSO model along with their LASSO coefficients are provided in Supplementary Table 4. The median pRiS value from D_1_ was −1.04 and was used for dividing patients into high- and low-pRiS groups. Clinical characteristics within the high- and low-pRiS groups are summarized in Supplementary Table 5. In D_1_, there were significant differences in OS (Fig. 1a) comparing patients with high- versus low-pRiS. The prognostic performance of pRiS was confirmed on D_2_ and D_3_ for OS (D_2_, HR = 2.14, 95% CI, 1.1–4.16, *p* = 0.025, C-index = 0.6, Fig. 1b; D_3_, HR = 2.74, 95% CI, 1.34–5.62, *p* = 0.006, C-index = 0.64, Fig. 1c). For DFS, the difference between high- and low-pRiS groups were significant on D_1_ (Fig. 1d) but not significant on D_2_ and D_3_, although we could observe a clear separation of the KM curves (D_2_, HR = 1.81, 95% CI, 0.97–3.37, *p* = 0.06, C-index = 0.62, Fig. 1e; D_3_, HR = 1.69, 95% CI, 0.93–3.05, *p* = 0.08, C-index = 0.61, Fig. 1f). On D_2_, the 5-year OS and DFS rates were 80.0% and 78.8% for the high-pRiS patients, and 90.2% and 84.1% for the low-pRiS patients. On D_3_, the 5-year OS and DFS rates were 88.9% and 85.0% for the high-pRiS patients, and 91.9% and 91.4% for the low-pRiS patients. Intratumoral and peritumoral feature expression heatmaps for one example high-pRiS and one low-pRiS patient are provided in Fig. 2. Two features that contribute to construction of pRiS (Intratumoral Laws texture and peritumoral CoLlAGe inertia) were spatially mapped on top of the tumor itself and annular ring area around the tumor, with blue and red representing low and high feature values, respectively.

Multivariate Cox analysis on OS in D_2_ and D_3_ are provided in Table 2 and Supplementary Table 6, adjusting for clinical variables including age, gender, tumor subsite, smoking PY, T-stage, N-stage and treatment types. pRiS remained an independent prognostic factor for OS in D_1_ (HR = 25.5, 95% CI, 4.68–138.9, *p* = 0.0002), in D_2_ (HR = 2.24, 95% CI, 1.05–4.76, *p* = 0.04) and in D_3_ (HR = 7.59, 95% CI, 1.37–42.14, *p* = 0.02). Point-biserial correlation analysis between each individual prognostic radiomic feature including pRiS and the clinicopathologic factors are provided in Supplementary Table 7.

**Table 2:** Multivariable analysis on OS using the combined cohorts of D_2_ + D_3_.

Parameter	HR (95% CI)	*p* Value
Age	1.03 (1–1.06)	0.1
Gender		
Female	Ref	
Male	1.38 (0.65–2.94)	0.41
Tumor subsite		
Base of tongue	Ref	
Tonsillar complex	0.82 (0.46–1.46)	0.53
Posterior wall/soft palate	0.2 (0.04–2.18)	0.15
Smoking PY	1.02 (1.01–1.03)	**<0.0001**
T-stage		
T1	Ref	
T2	1.91 (0.85–4.34)	0.12
T3	2.67 (1.15–6.21)	**0.02**
N-stage		
N0	Ref	
N1	1.58 (0.64–3.89)	0.08
N2	1.44 (0.44–4.75)	0.15
Treatment		
Radiotherapy	Ref	
Chemoradiation	0.42 (0.24–0.73)	**0.002**
pRiS	3.45 (1.09–10.96)	**0.03**

Note: bold values refer to statistically significant by two-tailed test, *p* < 0.05.

*OS* overall survival, *HR* hazard ratio, *PY* pack-year, *CI* confidence interval.

### Incremental prognostic value of pRiS

**Fig. 3:**
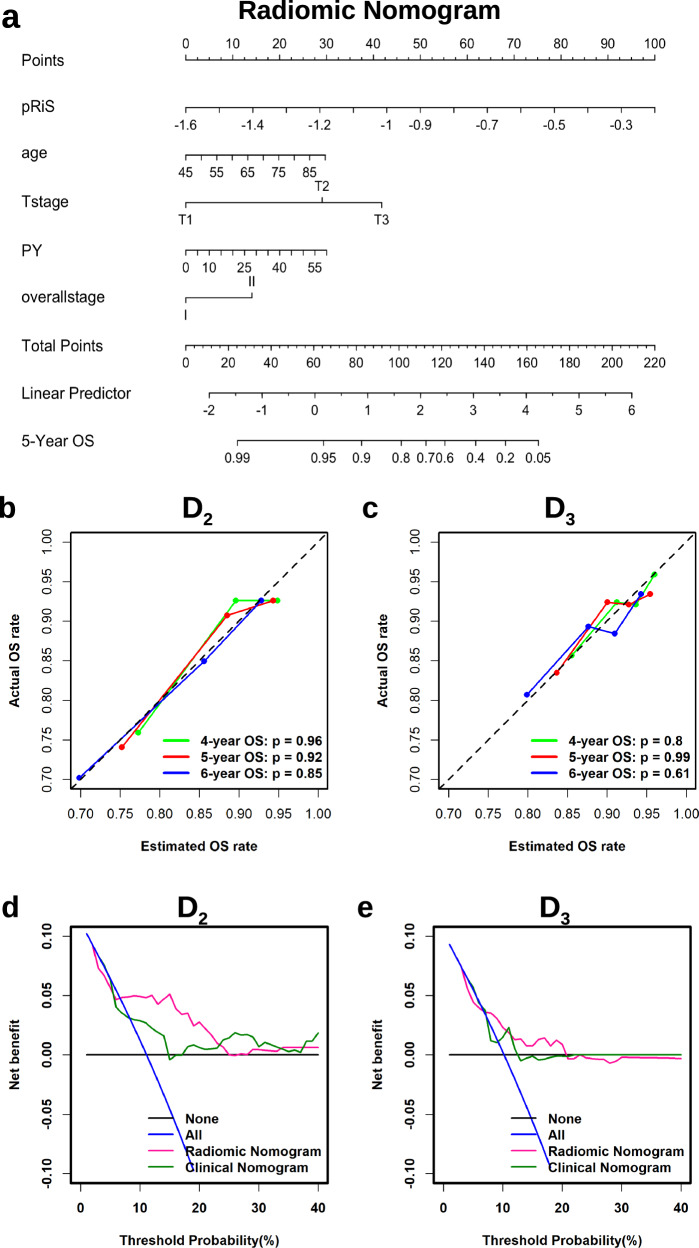
The radiomic nomogram and its calibration and decision curve analysis. The nomogram consists of pRiS, age, T-stage, smoking PY, and AJCC 8th staging (**a**). Calibration curves showed good agreement between the predicted and actual OS on D_2_ (**b**) and D_3_ (**c**). Decision curve analysis demonstrated higher net benefit of OS predictions on D_2_ (**d**) and D_3_ (**e**) from the radiomic nomogram M_rad+c_ compared to the clinical nomogram M_c_. Net benefit = true positive rate − (false positive rate × weighting factor). Weighting factor = Threshold probability/1 − threshold probability.

pRiS, age, smoking PY, T-stage and AJCC overall stage were found to be significantly associated with OS in univariate Cox analysis in D_1_ (Supplementary Table 8) and were incorporated into the M_rad+c_ (Fig. 3a). The clinical nomogram M_c_ is provided in Supplementary Fig. 1. The calibration curves for the radiomic nomogram M_rad+c_ at 4, 5 and 6 years showed good agreement between the actual and the estimated OS (Fig. 3b, c) and DFS (Supplementary Fig. 2) on D_2_ and D_3_. The decision curve analysis for OS prediction revealed that the M_rad+c_ had higher clinical net benefit than M_c_ when the threshold probabilities are less than 20% (Fig. 3d, e). M_rad+c_ resulted higher C-index compared with M_c_ regarding OS (0.68 vs 0.64, *p* = 0.06) and DFS (0.6 vs 0.57, *p* = 0.1) estimation (Table 3), although the differences were not significant. We then obtained the prognostic accuracy of pRiS, M_rad+c_ and M_c_ using time-dependent ROC analysis at specific times for predicting 4-, 5-, and 6-year OS and DFS. We observed consistently higher AUC from M_rad+c_ compared with M_c_ across D_1_, D_2_, and D_3_ for both survival endpoints (Supplementary Fig. 3).

**Table 3:** C-indices of nomograms.

Models	D_1_		D_2_ + D_3_	
	OS	DFS	OS	DFS
M_rad+c_	0.80	0.77	0.68	0.60
M_c_	0.72	0.69	0.64	0.57

C-indices of the radiomic nomogram (M_rad+c_) and the clinical nomogram (M_c_) on OS and DFS estimation.

### pRiS for predicting chemotherapy benefit

**Fig. 4:**
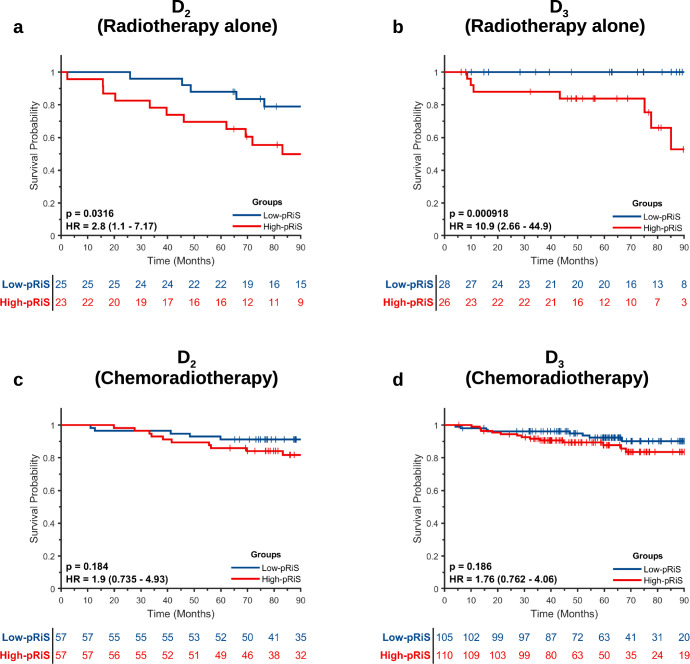
Stratified Kaplan–Meier survival analysis according to treatment arms between high-pRiS and low-pRiS groups (using median cutoff). On both D_2_ and D_3_, the high-pRiS groups have significant worse OS than the low-pRiS group when treated with radiotherapy alone (**a**, **b**) while the separations in the chemoradiation arm (**c**, **d**) were not significant, indicating high-pRiS patients potentially could benefit from chemotherapy.

For patients treated with only radiotherapy, high-pRiS patients had significantly worse OS compared to low-pRiS patients in both D_2_ (HR = 2.8 [1.1–7.17], *p* = 0.0316, Fig. 4a) and D_3_ (HR = 10.9 [2.66–44.9], *p* = 0.0009, Fig. 4b). By contrast, no significant differences in OS were observed between the high-pRiS and the low-pRiS patients treated with chemoradiation in either D_2_ (HR = 1.76 [0.76–4.06], *p* = 0.186, Fig. 4c) or D_3_ (HR = 1.9 [0.735–4.93], *p* = 0.184, Fig. 4d). These consistent patterns across D_2_ and D_3_ suggest that only the high-pRiS patients were able to derive benefit from chemotherapy, as reflected by their better OS when treated with chemotherapy in addition to radiation.

**Fig. 5:**
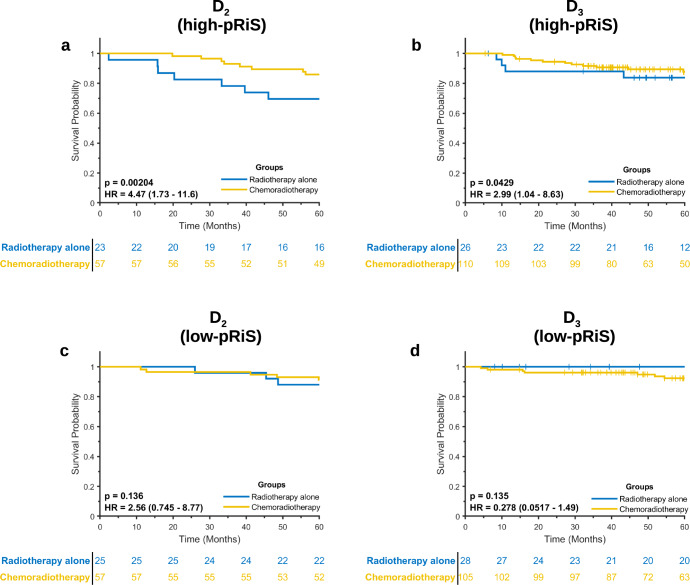
Kaplan–Meier survival analysis for comparing OS between patients treated with radiotherapy alone and treated with chemoradiotherapy. Only high-pRiS patients did benefit from chemotherapy (**a**, **b**) while there was no advantage or potential negative impact on survival in low-pRiS patients (**c**, **d**) when treated with chemotherapy.

We then compared OS between patients in the two treatment arms and found that only high-pRiS patients tended to have longer survival when chemotherapy was administered while low-pRiS patients did not. For both D_2_ and D_3_, chemotherapy was associated with an improved OS in high-pRiS patients (radiation vs chemoradiation, D_2_, HR = 4.47 [1.73–11.6], *p* = 0.002, Fig. 5a; D_3_, HR = 2.99 [1.04–8.63], *p* = 0.0429, Fig. 5b). On the other hand, for patients in the low-pRiS group, chemotherapy did not affect OS (radiation vs chemoradiation, D_2_, HR = 2.56 [0.745–8.77], *p* = 0.136, Fig. 5c; D_3_, HR = 0.278 [0.052–1.49], *p* = 0.135, Fig. 5d). We also performed the experiments using DFS as the endpoint and obtained similar results (Supplementary Fig. 4 and Fig. 5). We also used the threshold output −1.1 (with the smallest *p* value in D_1_) from X-tile software as the cutoff to define the pRiS groups and repeated the predictive experiments for OS (Supplementary Figs. 6 and 10) and DFS (Supplementary Fig. 7) as outcomes. When combining D_2_ and D_3_ for interaction test, there was a significant interaction between treatment and pRiS groups in the Cox regression model (*p* = 0.04), indicating a predictive effect of pRiS. On subset analysis by AJCC 8th overall stage (stage I and II), pRiS was predictive of chemotherapy benefit for stage II (*p* = 0.047; Supplementary Fig. 8) but not for stage I patients (*p* = 0.38; Supplementary Fig. 9).

## Discussion

The European Organization for Research and Treatment of Cancer (EORTC) 22931^10^ and Radiation Therapy Oncology Group (RTOG) 95-01 randomized clinical trial^6^ demonstrated the survival benefit of concurrent chemoradiotherapy in high-risk head and neck cancer patients. This paved the way for chemoradiotherapy to become the standard treatment protocol^22,23^ for locally advanced OPSCC patients, regardless of HPV status. However, clinical characteristics and treatment response differ between various head and neck cancer subsites^24^ (e.g., oropharyngeal, laryngeal and oral cavity). As the HPV-associated OPSCCs have more favorable prognosis and are more responsive to radiation than the HPV negative OPSCC^2^, it is likely that some of these patients do not receive added benefit from chemotherapy and might have had a similar outcome if treated with radiotherapy alone. With the advent of HPV related cancers causing a significant epidemiological shift, there is a growing call for more specific and selective treatment planning strategies to be reconsidered for HPV-associated OPSCC^12,22^.

Currently, there are many treatment de-escalation strategies for HPV-associated OPSCC patients, including eliminating chemotherapy or reducing the dosage of radiotherapy, with no consensus on an optimal choice. Recently, a randomized phase II trial (NRG-HN002)^12^ assigned HPV-associated OPSCC patients into one of two de-escalated treatment arms: (1) reduced dosage of radiotherapy with weekly cisplatin, or (2) accelerated radiotherapy (60 Gy) alone. Results showed that there were no significant differences (*p* = 0.23) in progression-free survival (PFS) rate between the two arms and the two-year OS rates were similar (96.7% and 97.3%). These results potentially indicate that not all selected candidates for treatment de-escalation benefited from the additional chemotherapy and they could have avoided the aggressive chemotherapy without compromising outcome. The interim analysis of NRG HN-005 trial failed to demonstrate the non-inferiority of 60 Gy of radiation plus cisplatin arm to the standard arm of 70 Gy with cisplatin. One possible explanation to this preliminary result is that the current selection criteria for lower risk HPV + OPSCC patients, which is based on clinical T/N stages and smoking status, remains insufficient for risk stratification. Development of novel and robust biomarkers to improve the accuracy of prognostication for HPV + OPSCC patients may aid in the design of future de-intensification trials. While definitive chemoradiation differs from the adjuvant treatment protocols, it still highlights the therapeutic importance of chemotherapy. Given these results, an individualized approach to accurately identify OPSCC patients who are most likely to benefit from chemotherapy would enable delivery of precision care to these patients.

In this work, we present a prognostic CT-based radiomic signature (pRiS) which is also predictive of added benefit of chemotherapy using a cohort of 491 AJCC 8th edition stage I and II HPV-associated OPSCC. pRiS comprises 7 radiomic textural features, one of which captures spots and waves textural heterogeneity patterns from within the primary tumor and six from the peritumoral region characterizing the tissue microenvironment around the tumor. Within the 0–15 mm annular ring around the tumor, 2 Gabor feature quantifying filter response and four CoLlAGe features characterizing co-occurrence of gradient orientations and intensity disorders were found to be prognostic. In multivariable analysis, pRiS was found to be an independent prognostic predictor and was able to stratify patients into high- and low-risk groups with significant differences in OS. Clinically, AJCC 8th edition stage I and stage II HPV-associated OPSCC patients could be considered for treatment de-escalation. However, a subset of these patients still had poor survival outcome and need to be identified for better treatment management^24,25^. The pRiS developed in this study was able to risk stratify these two populations, indicating that the true low-risk patients from these two clinically defined low-risk groups could be distinguished. On the other hand, high-pRiS patients were associated with worse outcome and thus should not be treated with de-escalation protocols. In addition, an integrated nomogram combining clinical factors and radiomic could improve the prognostic accuracy than using either alone^26,27^. These results indicate that pRiS could provide complementary information on OPSCC outcome beyond what is obtainable via currently known prognostic predictors.

pRiS was also found to be predictive of added benefit of chemotherapy for AJCC 8^th^ stage I and II HPV-associated OPSCC patients, which is the main innovative contribution of this work. To the best of our knowledge, there is no existing studies aimed to identify HPV-associated OPSCC patients who might not benefit from chemotherapy in addition to the definitive radiotherapy. Although previous work focused on risk-stratification and prognosis prediction, these studies could not provide an individualized treatment strategy based solely on the predicted risk profile^18–21^. In contrast, the pRiS developed in this study not only carry prognostic value but more importantly it could also convey which patients would derive additional benefit if treated with chemotherapy versus those who would not. This would enable a more granular and robust treatment de-escalation for OPSCC patient with low risk of recurrence. Currently, the criteria for selecting candidates suitable for chemotherapy are based on AJCC 7th edition staging^28–31^, which did not take HPV status into account for risk stratification. In our study, we found that patients with high-pRiS scores using median value from training set as cutoff (>−1.04) were estimated to benefit from chemotherapy with reduced hazard of dying while low-pRiS patients (<−1.04) showed non-significant hazard ratios between the two treatment arms when using OS as the endpoint. A slight change in the high- and low-pRiS membership using X-tile cutoff selection (−1.1) did not alter the statistical significance found in the radiotherapy alone cohorts, indicating the robustness of pRiS regarding predictive power (Fig. S10A, B). These results suggest that most of the high-pRiS patients in radiotherapy treatment arm are “truly high risk” patient populations with worse outcome and could have benefited from additional chemotherapy after radiation. This is strongly suggested by results in the chemoradiation arm, where we do not observe significant survival difference between high- and low-pRiS populations (Fig. S10C, D), potentially suggesting that high-pRiS patients have improved survival outcome due to receiving benefit from chemotherapy. When using DFS as the endpoint, high-pRiS patients derived statistically significant DFS benefit in D_2_ when treated with chemotherapy while did not yield the same significant DFS benefit in D_3_ (*p* = 0.074). However, we could observe a clear trend that patients who received chemoradiation had more favorable prognosis compared with those treated with radiation alone. Interestingly, low-pRiS patients in D_3_ showed detrimental effect of chemotherapy (HR < 1, radiotherapy alone vs chemoradiotherapy), regardless of the endpoint (OS, Fig. 5d; DFS, Fig. S5D). These results suggest chemotherapy only improved high-pRiS patients’ outcome and there is no added benefit of instituting potentially toxic chemotherapy for low-pRiS patients with favorable prognosis.

Machine learning-based approaches have been applied to radiographic images for prognosticating outcome for head and neck cancer patients. Ou et al.^32^ retrospectively analyzed 120 patients with locally advanced HNSCC and constructed a signature integrating radiomics with p16 status. They found that patients with high signature score significantly benefited from chemoradiation while no benefit from low signature patients was observed. While the results were promising, this study did not investigate the predictive value of peritumoral radiomic. In another study conducted by Howard et al.^33^, 33,527 HNSCC patients were included for analysis and chemotherapy recommendations from three deep learning (DL) models were all associated with survival benefits. It is known that DL-based approaches provide limited interpretability. On the other hand, the selected hand-crafted features in this study for pRiS includes descriptors characterizing CT pixel textural patterns (e.g., local intensity heterogeneity and microscale disorder in gradient orientation). Kann et al.^34^ validated a DL algorithm in identifying Extranodal extension (ENE) on pretreatment CT scans in 144 patients with HNSCC. They demonstrated that the algorithm’s diagnostic performance surpassed that of specialized radiologists with head and neck cancer experience. Although pathologic ENE is an indication for adjuvant treatment escalation, to the best of our knowledge, there has been no studies on developing predictive imaging biomarker which could guide OPSCC clinical treatment decision in the definitive setting. A previous study by Leijenaar et al. externally validated the prognostic value of intratumoral radiomic signatures in a cohort of 542 OPSCC^35^ (C-index = 0.63). Our study is different from Leijenaar’s study in that we focused only on OPSCC patients with low-risk profile (HPV-associated stage I and II patients). Since HPV+ and HPV- patients are clinically considered different tumor entities with distinct outcome and treatment response, it is more meaningful to risk-stratify these two patient populations separately. Rather than combining these two populations together for analysis like Leijenaar et al study, we focused on prognosis and treatment response prediction specifically for HPV+ patients.

Within the HPV+ population, currently the main treatment strategy is to provide de-escalated treatment protocols (i.e., less dosage of radiotherapy and potentially avoid the aggressive chemotherapy). Existing studies (including Leijenaar et al.^35^) either only constructing prognostic biomarker without investigating their association with treatment benefit^18–21^ or managed to quantify the treatment benefit for head and neck cancer with various disease subsites^32,33^ (oropharyngeal, laryngeal and oral cavity). Our study not only developed a prognostic radiomic biomarker which enabled individualized risk prediction of HPV-associated OPSCC patients, but also tackled a clinical-significant problem of identifying potential candidates not benefiting from chemotherapy and thus for whom such toxic treatments could be avoided.

This work is significantly different from previous related publications^16,18,20,21^ by (a) pRiS was shown to be not only prognostic for OPSCC patients’ outcome but also predictive to added benefits of chemotherapy; (b) rather than analyzing patients with different HNSCC subtypes, we specifically investigated the role of chemotherapy in the context of AJCC 8th stage I and II HPV-associated OPSCC patients.

We acknowledge that our study did have its limitations. First, this is a retrospective study subject to confounders and biases. Second, radiation doses and type of chemotherapy are not strictly controlled for the two treatment arms. Third, we did not manage to assess the intra-observer and inter-observer agreement of the radiomic features, which might cause selected features susceptible to variation of the tumor annotations. Fourth, the training set D1 has a relatively small sample size with 20% of events, potentially limiting its generalizability to a broader population with a more diverse demographic distribution. This is suggested by the C-index discrepancy between the training and the validation datasets. More data is needed to construct a predictive biomarker with better generalizability. Nevertheless, the findings in this study could guide future studies in the design of more robust predictive biomarkers for HPV-associated OPSCC treatment de-escalation. Future investigations, such as a prospective clinical trial with a large patient cohort aimed at comparing survival benefit between high-pRiS OPSCC patients treated with and without chemotherapy, would be necessary to demonstrate its clinical utility.

In summary, we have developed and validated a seven-feature prognostic and predictive signature for chemotherapy benefit in patients with HPV-associated OPSCC. Further work on larger populations will be needed to validate this preliminary biomarker. If prospectively validated, pRiS could serve as an inexpensive, tissue non-destructive prognostic and predictive companion diagnostic tool to identify patients most likely to be safely de-intensified.

## Methods

### Patients and outcome

**Fig. 6:**
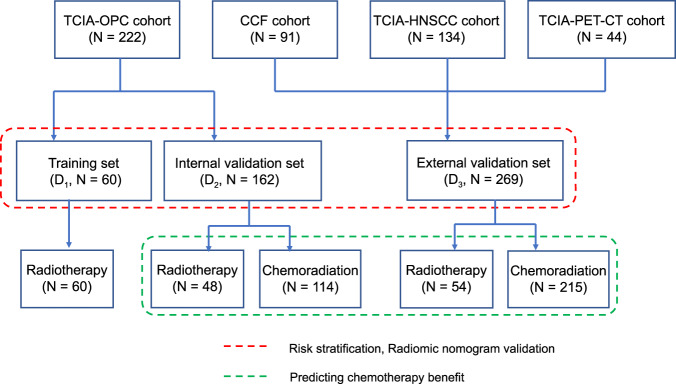
Patient selection diagram.

This retrospective study included 491 AJCC 8th edition stage I and II HPV-associated OPSCC patients from four different cohorts. Among the 222 patients from the Cancer Imaging Archive (TCIA)^36,37^ “OPC-Radiomic” cohort, 114 patients received chemoradiation and 108 patients received radiotherapy alone. 60 patients with radiotherapy alone from the “OPC-Radiomic” cohort formed the training set (D_1_) while the remaining 162 patients formed the internal validation cohort (D_2_). Among the 222 patients from the Cancer Imaging Archive (TCIA) “OPC-Radiomic” cohort, 114 patients received chemoradiation and 108 patients received radiotherapy alone. The fundamental rule is to unsure all patients in the training set received only radiation therapy (no chemotherapy), while maintaining descent number of patients treated with radiation alone in the internal validation set so as not to lose statistical power when comparing survival difference between the two treatment arms. We thought taking 60 out of the 108 patients treated with radiation alone to form the training set (D1) should be reasonable. Then all the remaining 48 radiation-treated patients plus 114 patients treated with chemoradiation (total 162) will be assigned to the internal validation set. We performed the following steps to determine the exact constitution of D1: we randomly selected 60 patients from the 108 patients treated with radiation alone and performed the statistical tests to see if there were statistically significant difference (*p* < 0.05) on clinicopathological variables (i.e., age, gender, AJCC 8th staging and disease recurrence) between the selected 60 patients and the remaining 162 patients (222-60). Differences between age were estimated using Wilcoxon rank-sum test and differences on gender, AJCC 8th staging, and disease recurrence were calculated using the chi-square test. We repeated this experiment for 500 iterations and documented the count for each patient which appears in iterations where there was no statistical difference between the selected 60 and the remaining 162 patients across all the four variables. Then the counts for all patients were re-sorted in descending order and we chose the top 60 patients to form the D1. The overall rationale is to keep balanced clinicopathological profile across the D1 and the D2. Three additional cohorts including cases from Cleveland Clinic Foundation (CCF, *N* = 91), TCIA-HNSCC (*N* = 134), and TCIA-Head-Neck-PET-CT (*N* = 44) were combined to form the external validation set (D_3_), which consisted of 269 patients in total. A patient selection diagram is provided in Fig. 6. HPV status for D_1_ and D_2_ cohorts were determined using p16 immunohistochemistry (IHC). A combination of p16 IHC and/or HPV DNA in situ hybridization were used for the D_3_ cohort. The waiver of written informed consent was granted by the IRB at Cleveland Clinic since all images are de-identified. In addition, it is not practicable to obtain consent from patients for this study since most patients was not alive or available for consent.

**Fig. 7:**
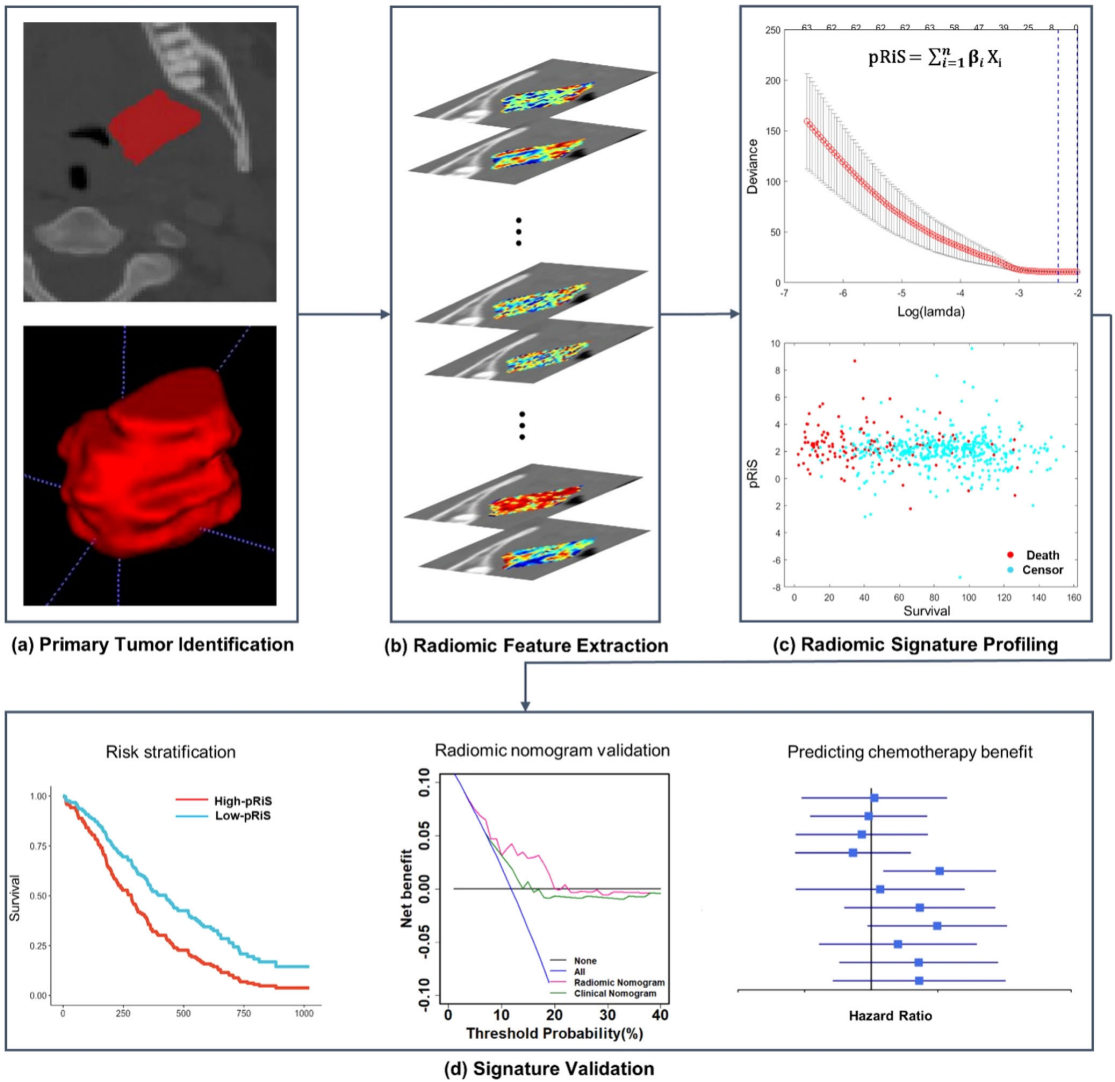
Flowchart of the radiomic workflow. **a** Primary tumor identification; **b** radiomic feature extraction; **c** radiomic signature profiling, and **d** signature validation.

Inclusion criteria were: (i) non-metastatic (M0) AJCC 8th stage I and II HPV-associated (strong and diffuse, block-like nuclear and cytoplasmic p16 staining present in ≥70% of the tumor specimen) OPSCC, (ii) with available baseline pretreatment CT images covering the head and neck region, (iii) treatment with curative intent, (iv) follow-up continued for at least 20 months or until death and (v) matched clinical information (e.g., age, stage and survival). Patients with any of the following were excluded: (i) lack of identifiable tumors on CT scans or (ii) number of pixels within tumor being less than 200, this was the minimum tumor volume that was deemed to be necessary for feature extraction. Disease-free survival (DFS) was defined as time from radiotherapy end date to the date of following events: local, regional, distant failure or death whichever occurred earlier and censored at the date of last follow-up for those without event. Overall survival (OS) was defined as the time from radiotherapy end date to death from any cause and censored at the date of last follow-up for those alive. Clinical and outcome information from patients in the CCF cohort was obtained by chart review after approval from the Institutional Review Board (IRB number: CCF 14–551). The overall workflow is provided in Fig. 7.

### CT image acquisition, segmentation, and compartment definition

All patients underwent an initial pretreatment CT scan with or without contrast agent injection for radiation therapy planning. CT images for the CCF cohort were acquired from GE (Chicago, IL) or Siemens (Erlangen, Germany) scanners. CT scans were acquired in helical mode with a slice thickness of 3 mm, at 120 kVp and 235 mAs tube current. Image resolution was between 0.4 mm - 0.5 mm for most of the patients, with image matrix of 512 × 512. The CT images for the three TCIA cohorts were acquired from one of the following CT scanners: General Electric Discovery ST; General Electric Lightspeed Plus; Toshiba Medical Systems Aquillion ONE. More details regarding the CT imaging parameters for the three TCIA cohorts included in this study could be found in Supplementary Table 1.

For primary tumor annotations on CCF cohort, two board-certified head and neck radiologists J.L. (with 5 years of clinical expertise) and S.S. (with 6 years of clinical expertise) manually delineated the tumor boundaries across all two-dimensional CT axial slices with visible tumor present using the 3D Slicer software^38^. The slices with dental artifacts were excluded for tumor annotations. The binary masks which comprise the outline of the gross primary tumor volume (GTV) for the three TCIA cohorts were obtained via the Radiation Therapy Structures (RTSTRUCT). RTSTRUCT is used to transfer anatomical structures related data between radiotherapy departments. It mainly comprises the information related to the regions of interest (ROIs) including GTV.

After obtaining the binary tumor mask for all patients, morphologic dilations were performed to define the annular ring region outside the tumor up to a radial distance of 15 mm based on a previous study^16^. The binary tumor masks were then subtracted from the dilated masks to obtain the peritumoral regions, which were then sub-divided into three peritumoral rings of 5-mm-radius increments. For all patients, peritumoral masks were dilated 15 mm from the corresponding intratumoral masks in a two-dimensional fashion. For each CT axial slice with tumor, the number of pixels dilated in each peritumoral mask were calculated as follows:

$${\rm{No}}.\;{\rm{of}}\;{{\rm{pixels}}}\;{{\rm{dilated}}}=\frac{15}{{{\rm{pixel}}}\;{{\rm{size}}}\;({{\rm{in}}}\;{{\rm{mm}}})}$$

During this peritumoral feature extraction process, additional consideration was taken to get rid of the region with air (<−900 HU). To avoid any edge artifacts that might arise during feature extraction, the filtered “dead” pixels of the CT scan were substituted by using an averaging filter across its 9 × 9 neighborhood.

### Radiomic feature extraction

Radiomic feature extraction was performed for each patient on the primary tumor using an in-house program developed with MATLAB 2020b (Mathworks, Natick, MA, USA), meeting IBSI criteria in terms of the reporting criteria for reproducible and transparent implementations. We resampled all CT images to an isotropic voxel size of 1 mm prior to feature extraction. The feature families utilized in this study included gray level intensity features, gray level co-occurrence matrix (GLCM) Haralick features^39^, Laws energy, Gabor wavelet-based features, and intensity gradient orientations features (CoLlAGe)^40^. These features were extracted on a per-pixel basis across all 2D slices with tumor for all patients from both intratumoral and peritumoral regions (0–5 mm, 5–10 mm, and 10–15 mm). The feature values were averaged across all slices. Statistics of mean, median, standard deviation (std), skewness, and kurtosis were calculated from the feature responses of all pixels within the region of interest, which resulted in a total of 2045 features. All feature values were transformed into new scores with a mean of 0 and a standard deviation of 1 (*z*-score transformation). Detailed description of extracted features is provided below:

#### Laws texture

2-dimensional Laws filters are derived by computing the outer product of combinations of the following 1-dimensional filter vectors focused on different texture patterns: Level (L5)—detects smoothness of intensity values, L5 = [1 4 6 4 1]; Edge (E5)—detects edges between regions with abrupt changes in intensity, E5 = [−1 −2 0 2 1]; Spot (S5)—detects speckled enhancement patterns, S5 = [−1 0 2 0 −1]; Wave (W5)—detects oscillating local intensity patterns, W5 = [−1 2 0 −2 1]; Ripple (R5)—detects oscillating intensity patterns centered at region of extreme intensity, R5 = [1 −4 6 −4 1].

To obtain a feature vector, convolution is performed on the filters and the images within a window size neighborhood followed by summing up all the values. Features are named by the combination of filters applied in the *y* and *x* axes, e.g., L5E5 is the product of a level detection filter in the y axis and an edge detection filter in the *x* axis.

#### Gabor filter responses

Two-dimensional Gabor filters are computed by modulating a Gaussian kernel function with one of 48 sinusoidal plane waves. Each sinusoidal plane wave corresponds to a unique combination of one of four spatial wavelengths (2 pixels, 4 pixels, 8 pixels, 12 pixels) and one of seven orientations (22.5°, 45°, 67.5°, 90°, 112.5°, 135°, 157.5°). Each Gabor filter is then convolved with the original image and values corresponding to filter response within the region of interest are concatenated.

#### Haralick features

The following Haralick gray level co-occurrence matrix (GLCM) descriptors were computed: entropy, energy, inertia, inverse difference moment, correlation, information measure of correlation 1, information measure of correlation 2, sum average, sum variance, sum entropy, difference average, difference variance, and difference entropy. GLCM statistics were concatenated within a window size neighborhood, yielding 13 descriptor vectors per region.

#### Co-occurrence of local anisotropy gradients (CoLlAGe)

An image’s intensity gradients in the *x* and *y* direction are computed. Within a window size neighborhood, the dominant intensity gradient orientation (between 0° and 360°) is computed via principal component analysis, resulting in a 2D array of equal size with the dominant gradient orientation value centered at the corresponding pixel of the original image. Metrics of the co-occurrence matrix are then applied to this gradient orientation image in the same manner as described above for Haralick GLCM features. The resulting 13 CoLlAGe descriptors quantify the homogeneity of intensity gradient directionality within an image.

### pRiS: a radiomic risk signature

The top prognostic radiomic features were selected by applying a least absolute shrinkage and selection operator (LASSO) Cox Proportional Hazard model^41^ on OS using D_1_. The optimal value of the tuning parameter in the LASSO Cox (alpha) model was determined by 10-fold cross validation to search for 100 values up to 1% of the estimated maximum. Once the parameter alpha is determined, the number of features was locked down to 7. pRiS was then constructed by linearly combining these 7 features weighted by their corresponding coefficients. The cutoff value for dividing patients into low- and high-risk patients was chosen using the median pRiS value from D_1_ based on OS. The potential association between the dichotomous pRiS group (high- vs low-pRiS group) and survival was first evaluated in D_1_ and then validated in D_2_ and D_3_ based on Kaplan-Meier (KM) survival analysis and Harrell’s concordance index (C-index).

### Combining pRiS with clinical factors to improve prognostication

To investigate whether pRiS adds incremental prognostic value to the clinical factors for individualized prediction of OS and DFS, we constructed an integrated radiomic nomogram (M_rad+c_) by combining pRiS with the prognostic clinical factors and compare it against a clinical nomogram (M_c_). Variables significant in univariate analysis in D_1_ were included in the M_rad+c_. Both the M_rad+c_ and M_c_ were developed in D_1_ based on the multivariable Cox regression analysis and tested in D_2_ and D_3_ with respect to calibration, discrimination (C-index), and clinical usefulness. Calibration curves along with the Hosmer–Lemeshow test were used to compare the predicted survival probabilities with the actual probabilities. Decision curve analysis was performed to quantify the net benefit at various threshold probabilities and to compare the clinical usefulness of the M_rad+c_ and M_c_.

### Association of pRiS with chemotherapy benefit

Patients from D_2_ and D_3_ sets who received either radiotherapy alone or chemoradiation were included for analysis. We evaluated pRiS in terms of its ability to predict the benefit of chemotherapy by comparing OS and DFS differences between patients who were treated with radiotherapy alone and those with chemoradiation in both the high-pRiS and low-pRiS groups. We also examined the predictive value of pRiS separately for AJCC 8th edition stage I and stage II patients. Furthermore, we used the cutoff values generated from the X-tile software^42^ version 3.6.1 to investigate the predictive value of pRiS on OS and DFS. X-tile provides a global assessment of every possible way of dividing a population into two groups based on the given biomarker, of which each possible biomarker value from the training set represents a unique cut-off point. The criteria to define the optimal threshold is to choose the cut-off point with the smallest p value from the log-rank test by iterating through all possible groupings defined by all unique cut-off points from training set. Using median value as the cut-off aims to maintain a balance between the number of patients in high- and low-pRiS groups in the training set while X-tile seeks to maximize the survival stratification between the two groups from a statistical perspective. The rationale for trying both cutoff-selection approaches is to investigate the robustness of predictive power carried by pRiS.

### Statistical analysis

The difference of the continuous variables (i.e., age, smoking pack-year [PY] and pRiS) among 3 datasets (D_1_–D_3_) was compared using the one-way analysis of variance test (ANOVA) and the association of the categorical variables with 3 datasets was estimated using the chi-square test. Differences of OS and DFS among groups was examined using the log-rank test. Univariate analysis of the continuous pRiS value with the clinical and pathologic factors (i.e., age, gender, tumor subsites, smoking in pack years, pathological T- and N-stages, and AJCC 8th overall stage) were conducted. Multivariable Cox regression analysis was performed to assess the relationships between the various clinical factors and OS. We also performed the point-biserial correlation analysis to investigate potential association between each individual prognostic radiomic feature and the clinicopathologic factors. Interaction between pRiS and chemotherapy was assessed by adding an interaction term in the Cox model. Statistical analyses were performed using R version 3.4.0. The R packages used in this study included glmnet, survminer, rms, survival, Hmisc, survMisc, survey, and SvyNom. A threshold of 0.05 was used to define statistical significance.

### Reporting summary

Further information on research design is available in the Nature Research Reporting Summary linked to this article.

## Supplementary information

REPORTING SUMMARY

Supplemental materials

## Data Availability

The institutional data underlying this article were provided by the Cleveland Clinic under license and permission. Data will be shared on request to the corresponding author with permission of the involved institution. The publicly available datasets could be accessed via following links: https://wiki.cancerimagingarchive.net/pages/viewpage.action?pageId=33948764; https://wiki.cancerimagingarchive.net/display/Public/HNSCC; https://wiki.cancerimagingarchive.net/display/Public/Head-Neck-PET-CT.

## Code availability

Both intratumoral and peritumoral feature extraction pipelines and the main scripts are provided at: https://github.com/BolinSong1995/NPJ_Precision_Oncology.
