# Supplementary material for: CT radiomic signature predicts survival and chemotherapy benefit in stage I and II HPV-associated oropharyngeal carcinoma
Source: NPJ Precis Oncol. 2023 Jun 2;7:53. doi: 10.1038/s41698-023-00404-w (PMC10238543; doi:10.1038/s41698-023-00404-w)
Supplement: Supplementary file 1 — REPORTING SUMMARY [file 41698_2023_404_MOESM1_ESM.pdf]

nature portfolio

Corresponding author(s): Anant Madabhushi

Last updated by author(s): 03/31/2023

Reporting Summary

Nature Portfolio wishes to improve the reproducibility of the work that we publish. This form provides structure for consistency and transparency in reporting. For further information on Nature Portfolio policies, see our Editorial Policies and the Editorial Policy Checklist.

Statistics

For all statistical analyses, confirm that the following items are present in the figure legend, table legend, main text, or Methods section.

n/a	Confirmed	
☐	☒	The exact sample size (*n*) for each experimental group/condition, given as a discrete number and unit of measurement
☐	☒	A statement on whether measurements were taken from distinct samples or whether the same sample was measured repeatedly
☐	☒	The statistical test(s) used AND whether they are one- or two-sided<br>*Only common tests should be described solely by name; describe more complex techniques in the Methods section.*
☐	☒	A description of all covariates tested
☐	☒	A description of any assumptions or corrections, such as tests of normality and adjustment for multiple comparisons
☐	☒	A full description of the statistical parameters including central tendency (e.g. means) or other basic estimates (e.g. regression coefficient) AND variation (e.g. standard deviation) or associated estimates of uncertainty (e.g. confidence intervals)
☐	☒	For null hypothesis testing, the test statistic (e.g. *F*, *t*, *r*) with confidence intervals, effect sizes, degrees of freedom and *P* value noted<br>*Give P values as exact values whenever suitable.*
☐	☐	For Bayesian analysis, information on the choice of priors and Markov chain Monte Carlo settings
☐	☒	For hierarchical and complex designs, identification of the appropriate level for tests and full reporting of outcomes
☐	☐	Estimates of effect sizes (e.g. Cohen's *d*, Pearson's *r*), indicating how they were calculated

Our web collection on statistics for biologists contains articles on many of the points above.

Software and code

Policy information about availability of computer code

Data collection	No software was used for data collection.
Data analysis	The feature extraction pipelines are publicly available at https://github.com/ccipd/scripts/tree/master/feature_extraction

For manuscripts utilizing custom algorithms or software that are central to the research but not yet described in published literature, software must be made available to editors and reviewers. We strongly encourage code deposition in a community repository (e.g. GitHub). See the Nature Portfolio guidelines for submitting code & software for further information.

Data

Policy information about availability of data

All manuscripts must include a data availability statement. This statement should provide the following information, where applicable:

- Accession codes, unique identifiers, or web links for publicly available datasets
- A description of any restrictions on data availability
- For clinical datasets or third party data, please ensure that the statement adheres to our policy

The institutional data underlying this article were provided by the Cleveland Clinic under license and permission. Data will be shared on request to the corresponding author with permission of the involved institution.

Human research participants

Policy information about studies involving human research participants and Sex and Gender in Research.

Reporting on sex and gender	Sex is considered in this study. Specifically, we conducted the multivariable analysis accounting for Sex in the Cox model.
Population characteristics	These information are provided in Table 1.
Recruitment	The inclusion criteria are: (i) non-metastatic (M0) AJCC 8th stage I and II HPV-associated (strong and diffuse, block-like nuclear and cytoplasmic p16 staining present in ≥70% of the tumor specimen) OPSCC, (ii) with available baseline pretreatment CT images covering the head and neck region, (iii) treatment with curative intent, (iv) follow-up continued for at least 20 months or until death and (v) matched clinical information (e.g. age, stage and survival).
Ethics oversight	Cleveland clinic

Note that full information on the approval of the study protocol must also be provided in the manuscript.

Field-specific reporting

Please select the one below that is the best fit for your research. If you are not sure, read the appropriate sections before making your selection.

☒ Life sciences ☐ Behavioural & social sciences ☐ Ecological, evolutionary & environmental sciences

For a reference copy of the document with all sections, see nature.com/documents/nr-reporting-summary-flat.pdf

Life sciences study design

All studies must disclose on these points even when the disclosure is negative.

Sample size	No sample size determinations were performed. We recruited all possible patients from institution and public cohort.
Data exclusions	Patients with any of the following were excluded: (i) lack of identifiable tumors on CT scans or (ii) number of pixels within tumor being less than 200, this was the minimum tumor volume that was deemed to be necessary for feature extraction.
Replication	The code and intermediate data could be provided to replicate the results.
Randomization	The allocation of data (training vs test sets) was performed by cohort sources, either by institutions or by public cohort from original sources. Allocation of risk group was calculated by the machine learning pipeline.
Blinding	We defined clear and objective criteria on how to divide the patients into risk groups, although we are not blinded to the grouping as this is a retrospective study.

Reporting for specific materials, systems and methods

We require information from authors about some types of materials, experimental systems and methods used in many studies. Here, indicate whether each material, system or method listed is relevant to your study. If you are not sure if a list item applies to your research, read the appropriate section before selecting a response.

Materials & experimental systems

n/a	Involved in the study	
☒	☐	Antibodies
☒	☐	Eukaryotic cell lines
☒	☐	Palaeontology and archaeology
☒	☐	Animals and other organisms
☐	☒	Clinical data
☒	☐	Dual use research of concern

Methods

n/a	Involved in the study	
☒	☐	ChIP-seq
☒	☐	Flow cytometry
☒	☐	MRI-based neuroimaging

Clinical data

Policy information about clinical studies

All manuscripts should comply with the ICMJE guidelines for publication of clinical research and a completed CONSORT checklist must be included with all submissions.

Clinical trial registration	na
Study protocol	na
Data collection	we collected data from collaborating institution CCF and public website TCIA
Outcomes	Overall survival (OS) was defined as the time from radiotherapy end date to death from any cause and censored at the date of last follow-up for those alive.
