# Supplementary material for: CT radiomic signature predicts survival and chemotherapy benefit in stage I and II HPV-associated oropharyngeal carcinoma
Source: NPJ Precis Oncol. 2023 Jun 2;7:53. doi: 10.1038/s41698-023-00404-w (PMC10238543; doi:10.1038/s41698-023-00404-w)
Supplement: Supplementary file 2 — Supplemental materials [file 41698_2023_404_MOESM2_ESM.docx]

**Supplemental Material**


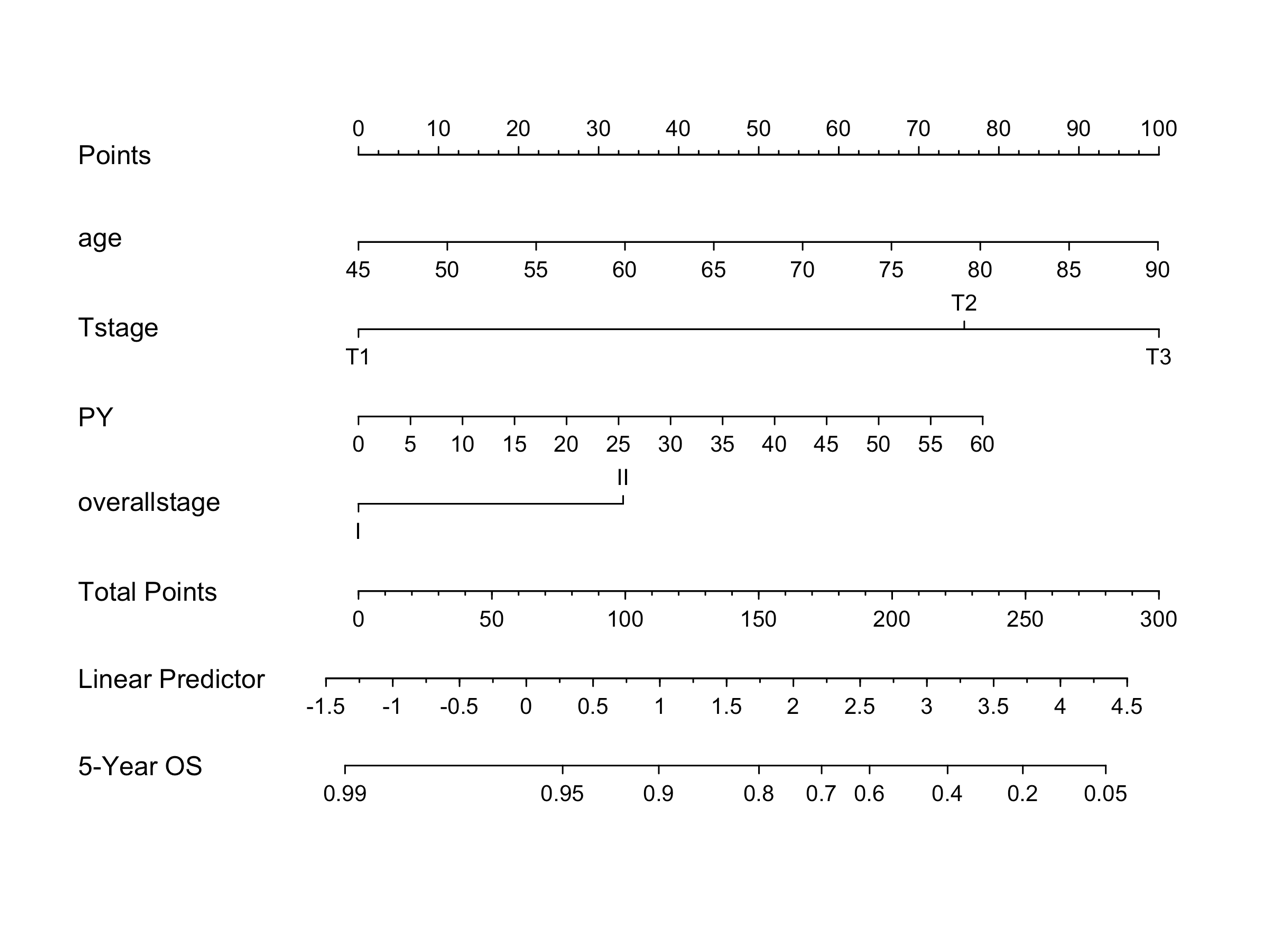


**Supplementary Figure 1. The clinical nomogram (M_c_) constructed in this study.**


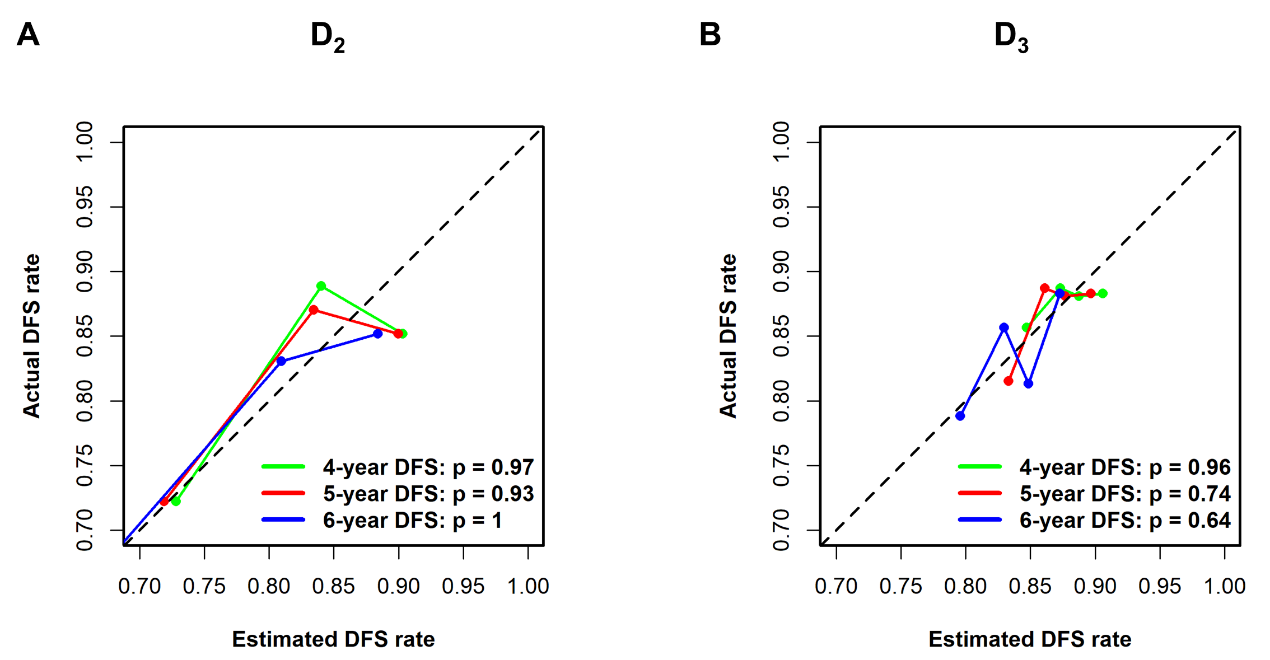


**Supplementary Figure 2. Calibration curves using DFS cutoff at 4, 5 and 6 years.** There are good agreements between the predicted and actual DFS on D2 (A) and D3 (B).


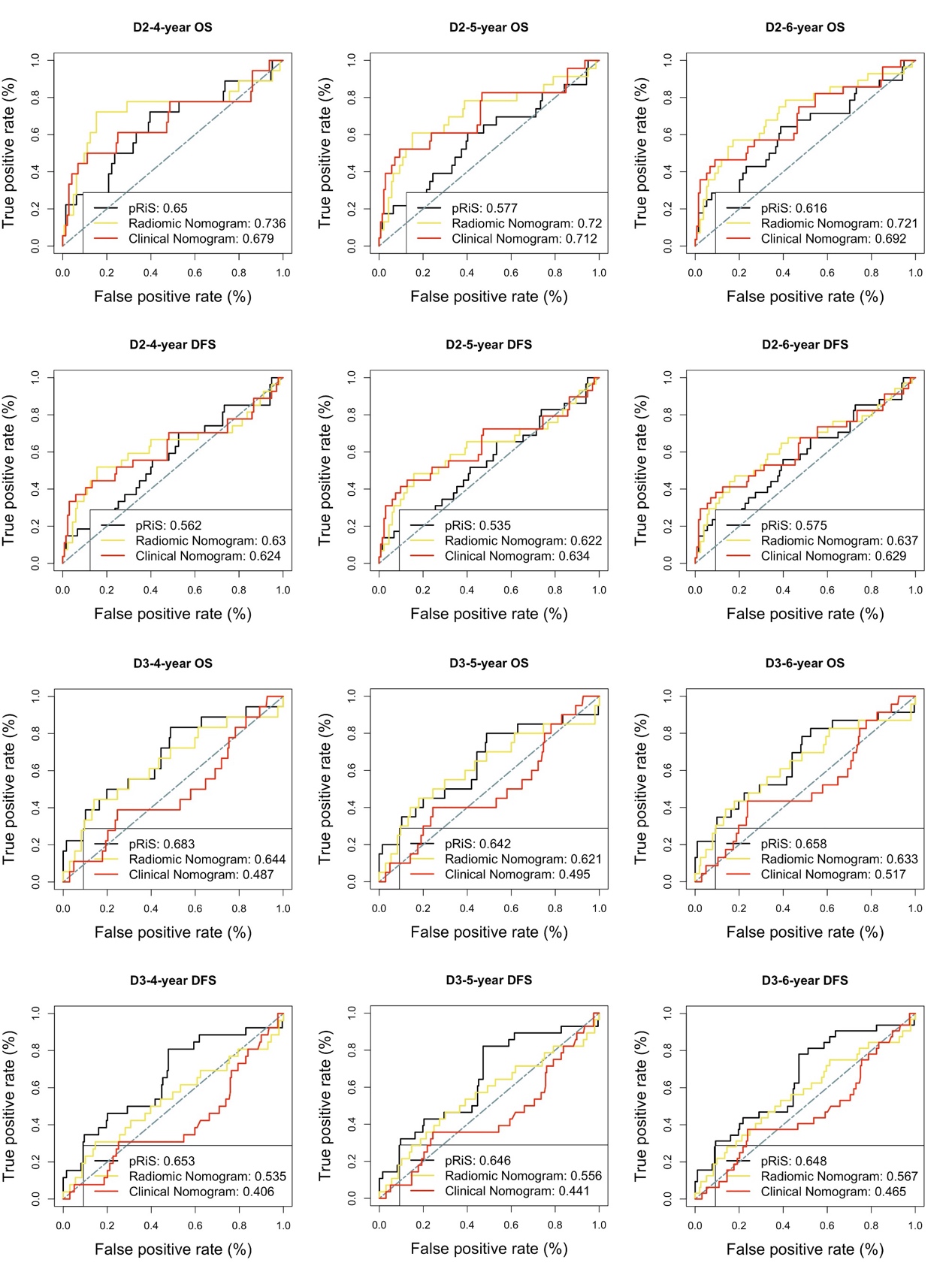


**Supplementary Figure 3. Time-dependent ROC curves**. Predictions are made to show the 4-, 5- and 6-year overall survival (OS) on D2 (first row) and D3 (third row), and disease-free survival (DFS) on D2 (second row) and D3 (fourth row). Prediction accuracies are provided using pRiS, the radiomic nomogram (M_rad+c_) and the clinical nomogram (M_c_). M_rad+c_ had higher AUC than M_c_ in most of the survival endpoints.


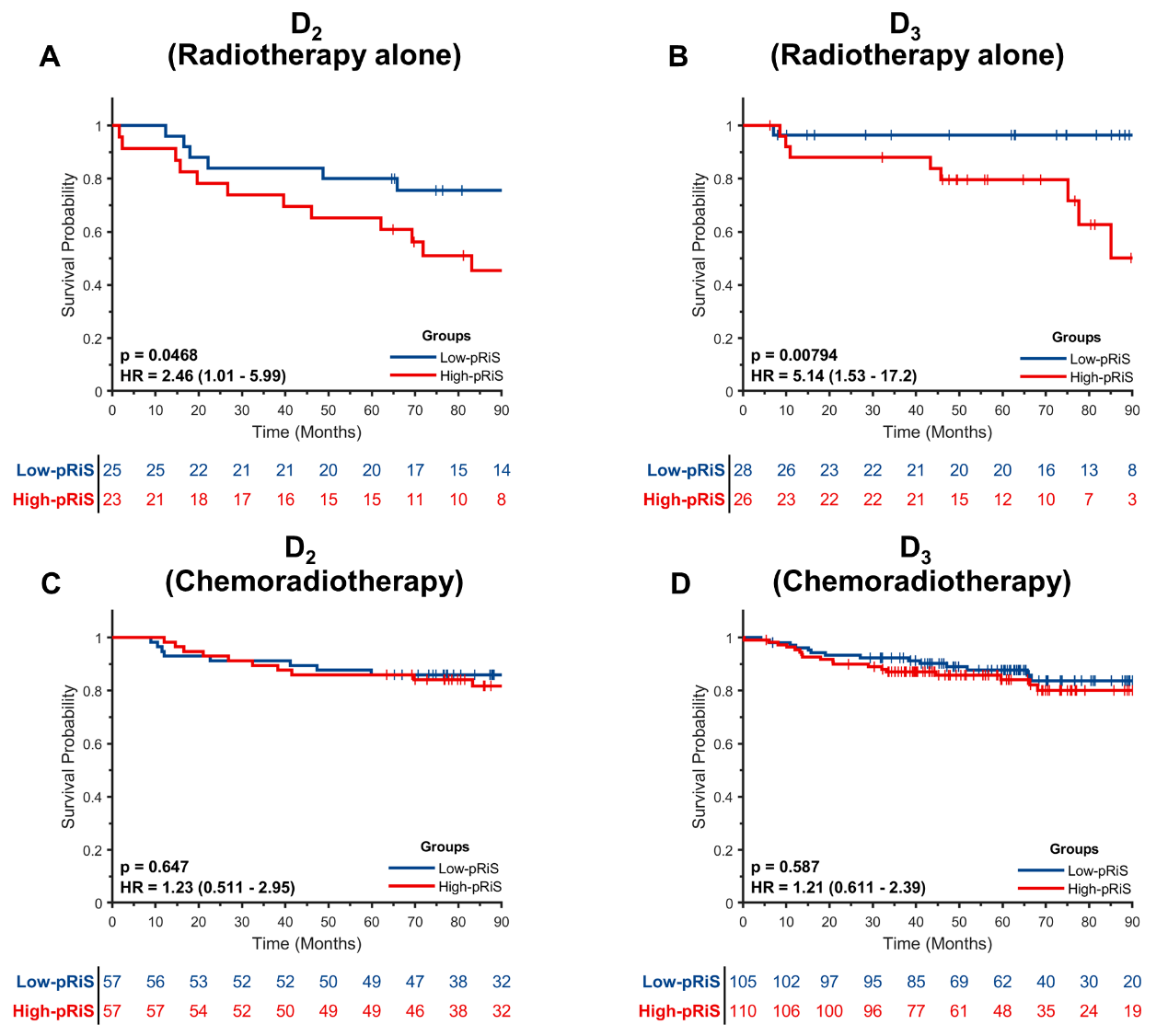


**Supplementary Figure 4.** **Stratified Kaplan-Meier survival analysis according to treatment arms using DFS as endpoint**. On both D2 and D3, the high-pRiS groups have significant worse DFS than the low-pRiS group when treated with radiotherapy alone while the separations in the chemoradiation arm were not significant, indicating high-pRiS patients potentially could benefit from chemotherapy.


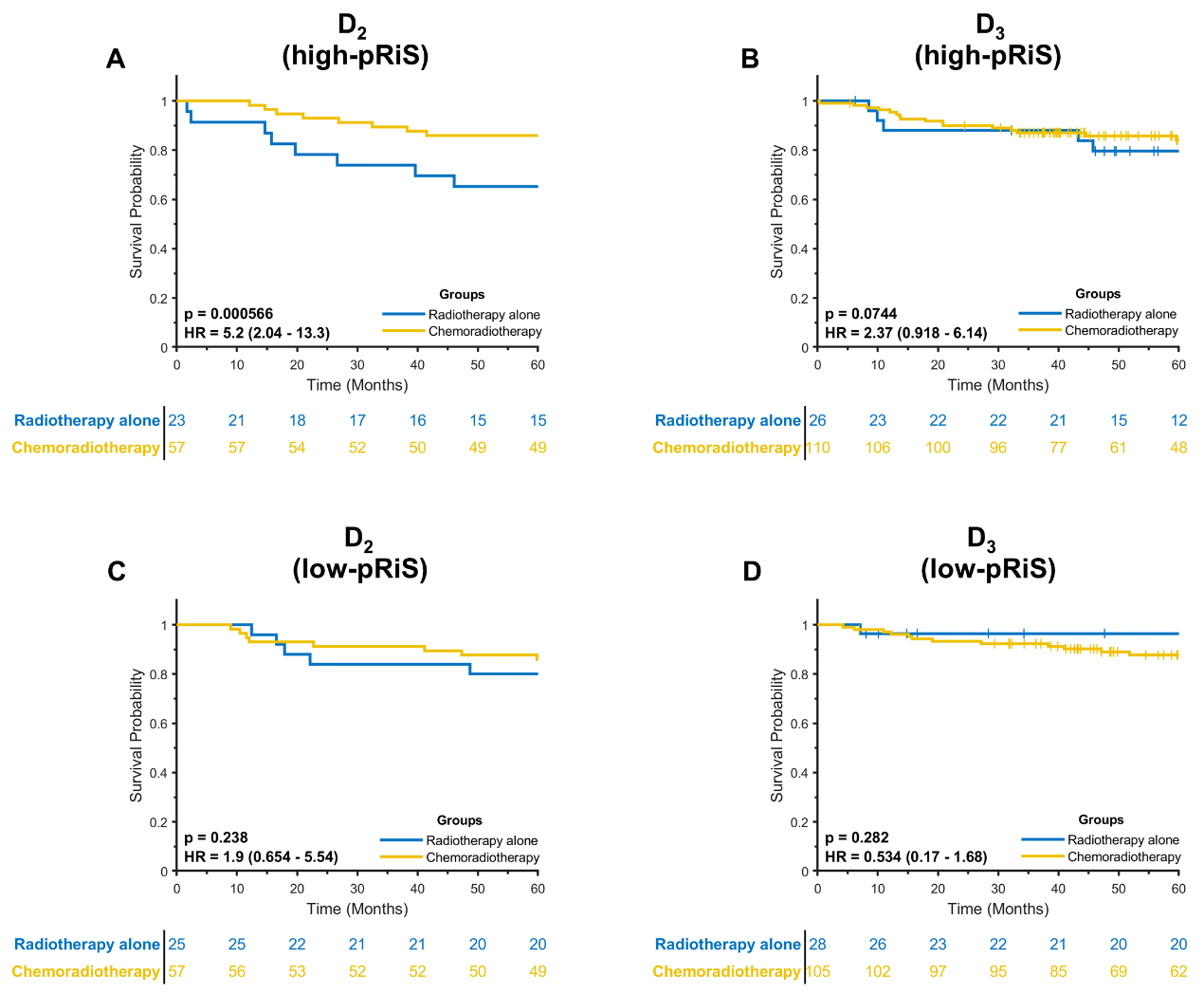


**Supplementary Figure 5. Kaplan-Meier survival analysis for comparing DFS between patients treated with radiotherapy alone and treated with chemoradiotherapy**. Only high-pRiS patients did benefit from chemotherapy (A, B) while there was no advantage or potential negative impact on DFS in low-pRiS patients (C, D) when treated with chemotherapy.


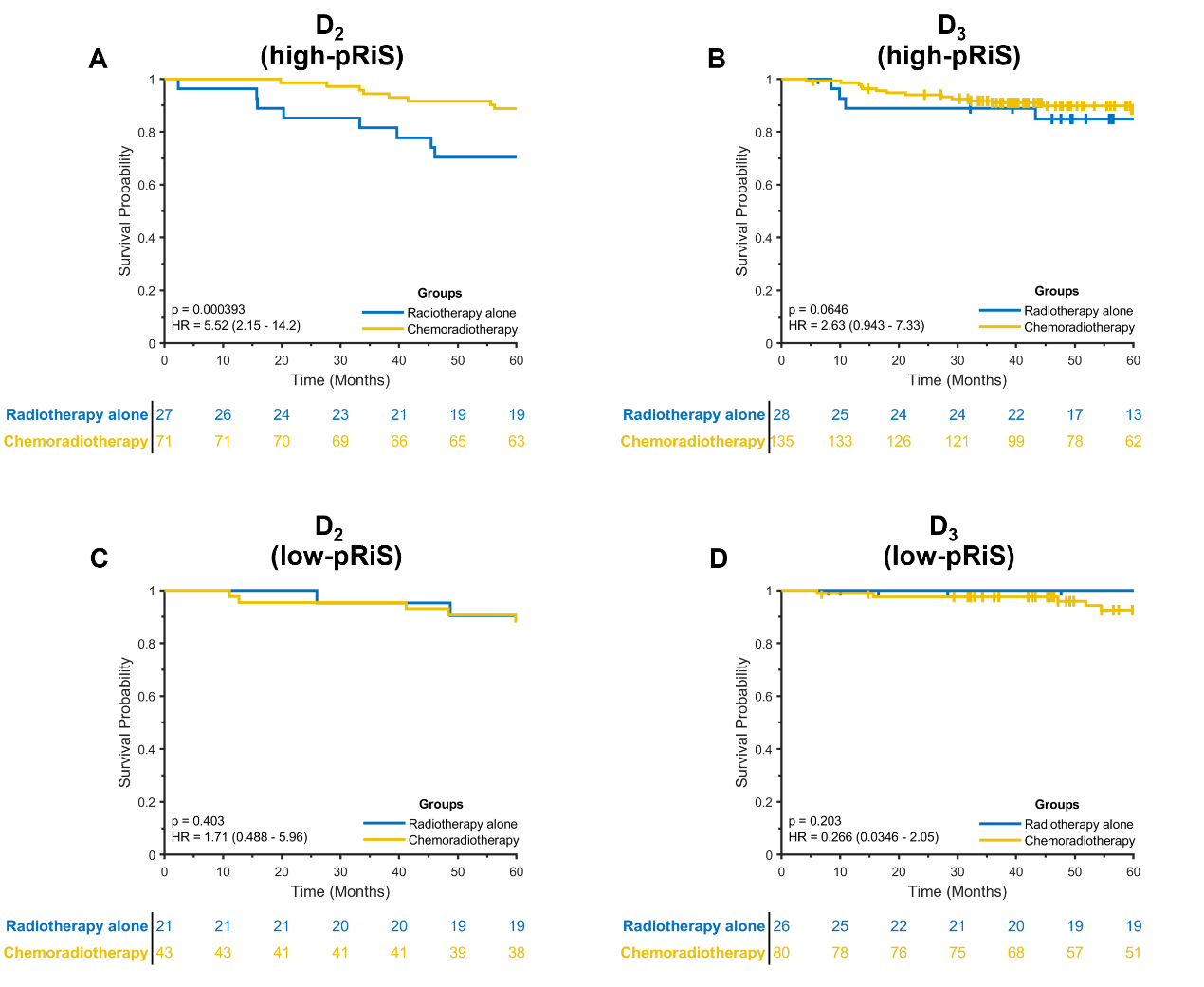


**Supplementary Figure 6. Kaplan-Meier survival analysis for comparing OS between treatment arms** **using cutoff threshold output -1.1 from the X-tile software.** The predictive value of the pRiS still exists after using the new threshold to define the high- and low-pRiS groups.


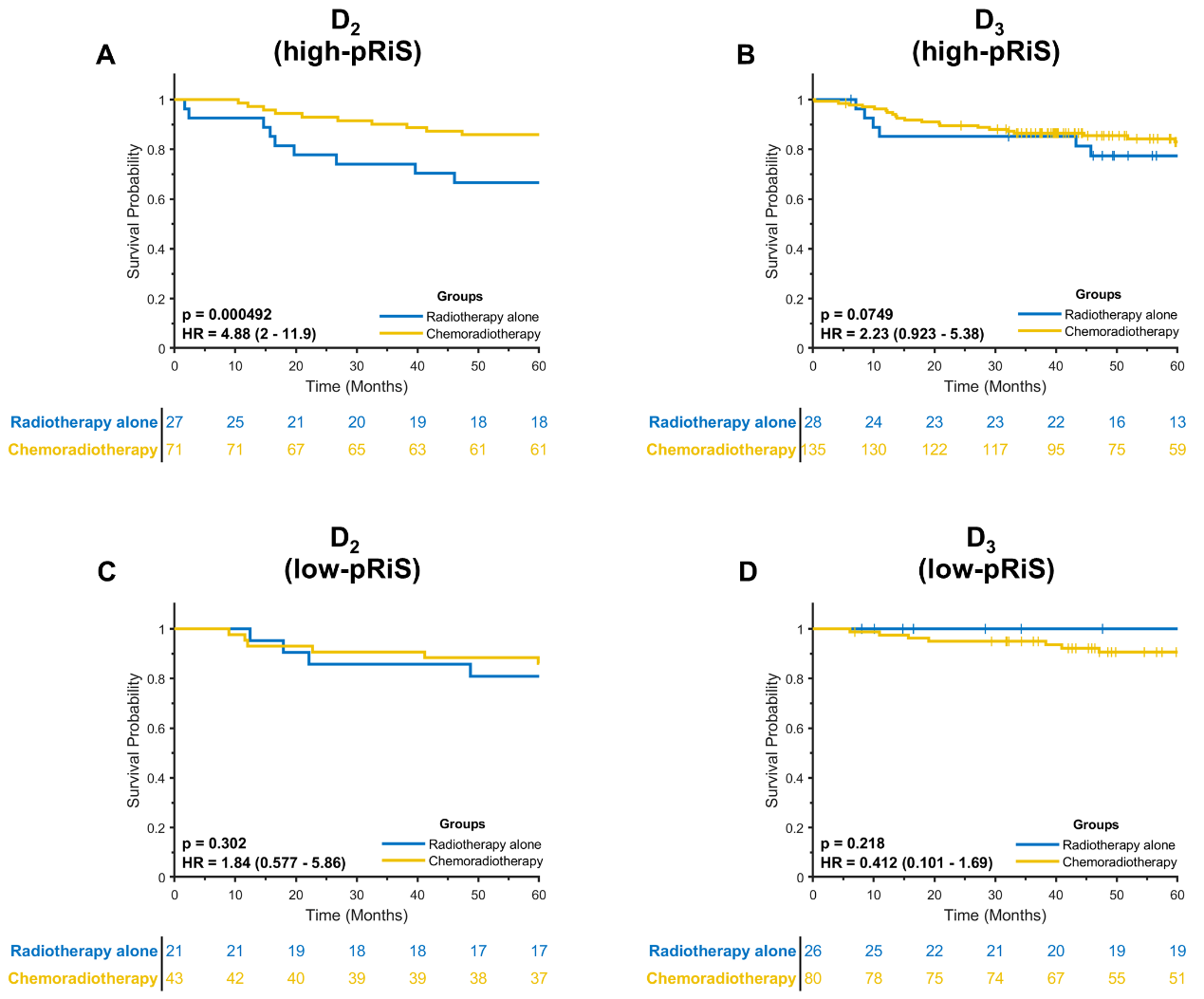


**Supplementary Figure 7. Kaplan-Meier survival analysis for comparing DFS between treatment arms** **using cutoff threshold output -1.1 from the X-tile software.** The predictive value of the pRiS still exists after using the new threshold to define the high- and low-pRiS groups.


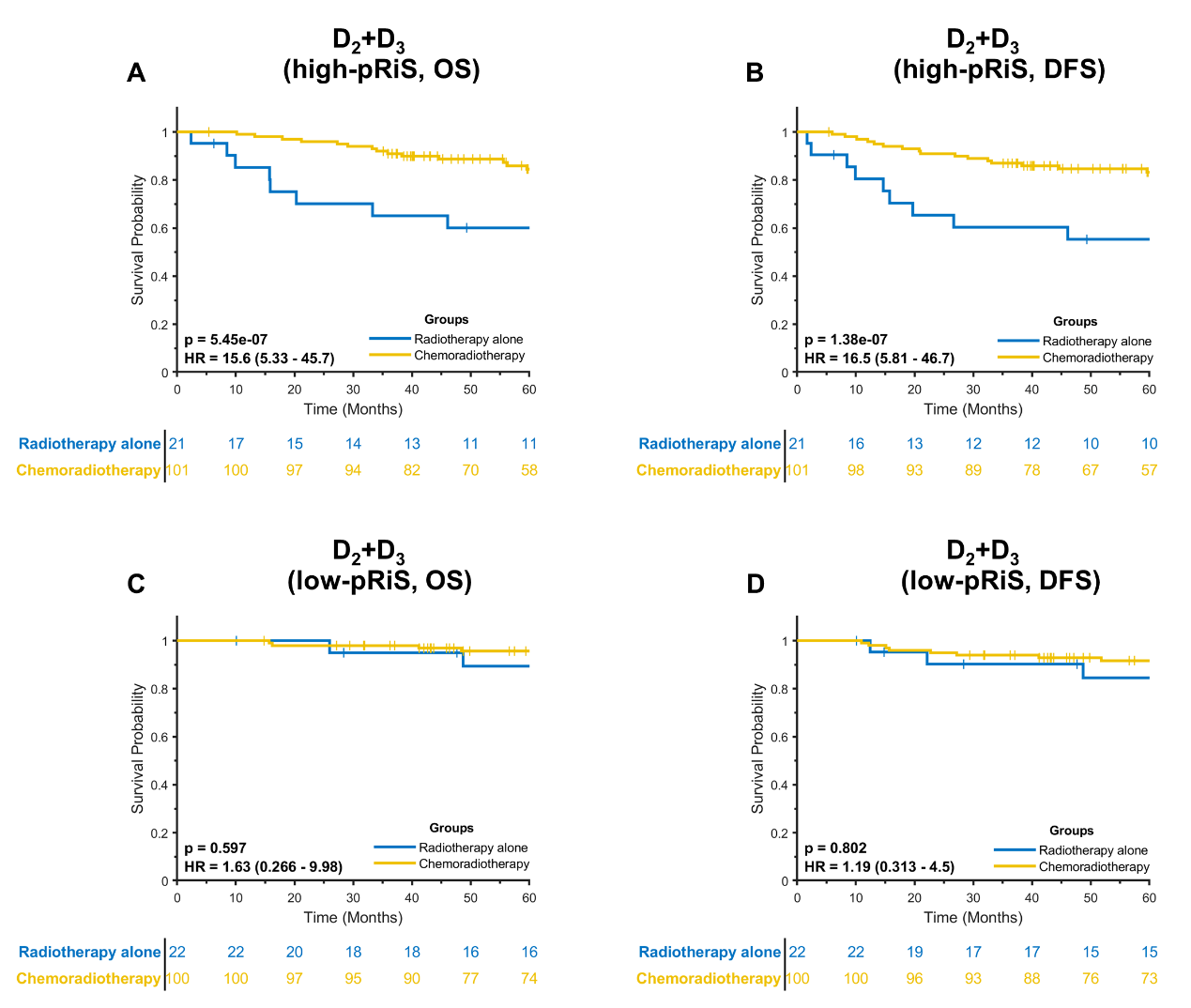


**Supplementary Figure 8. Kaplan-Meier survival analysis for comparing OS between treatment arms for AJCC stage II patients only.**


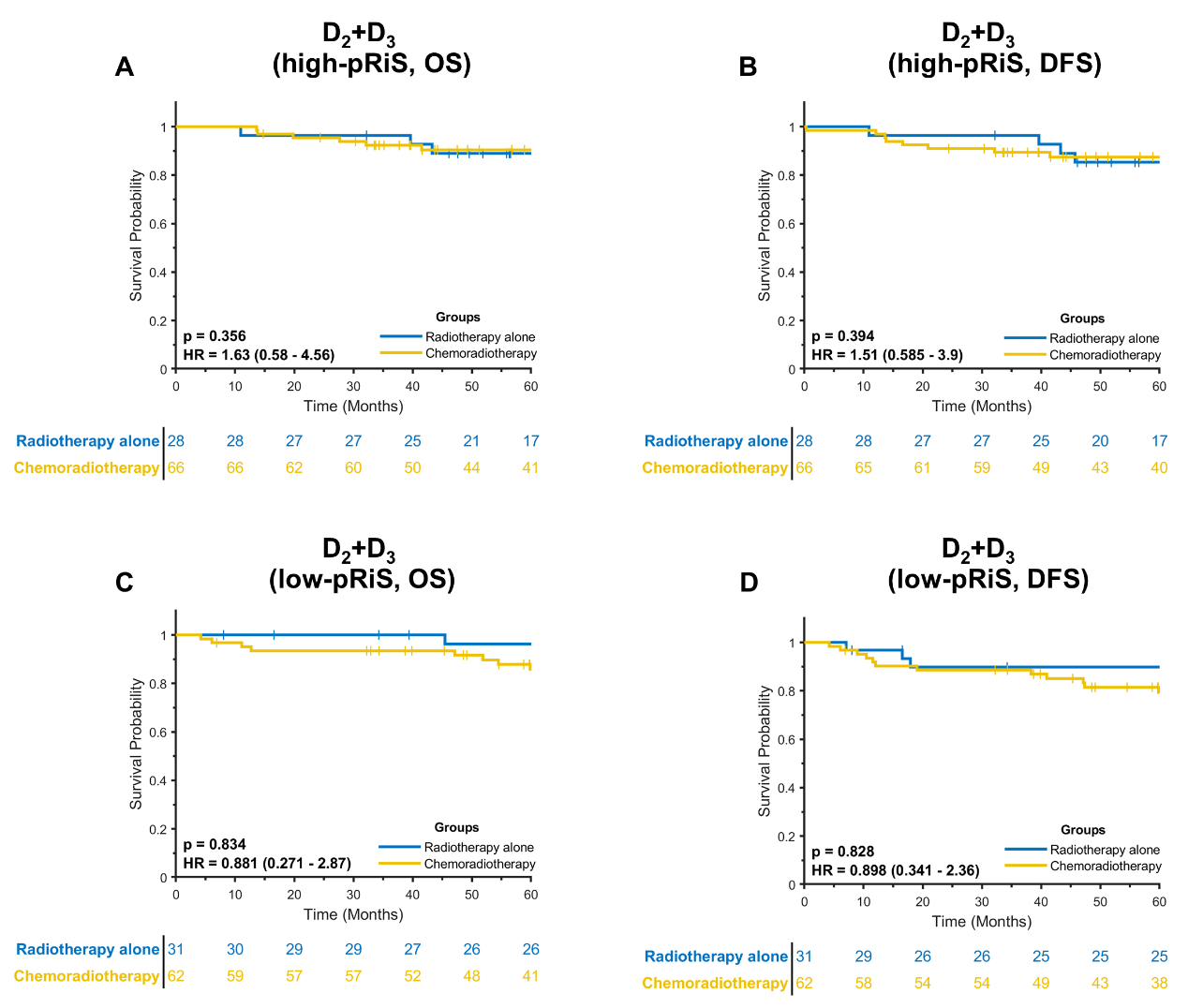


**Supplementary Figure 9. Kaplan-Meier survival analysis for comparing OS between treatment arms for AJCC stage I patients only.**


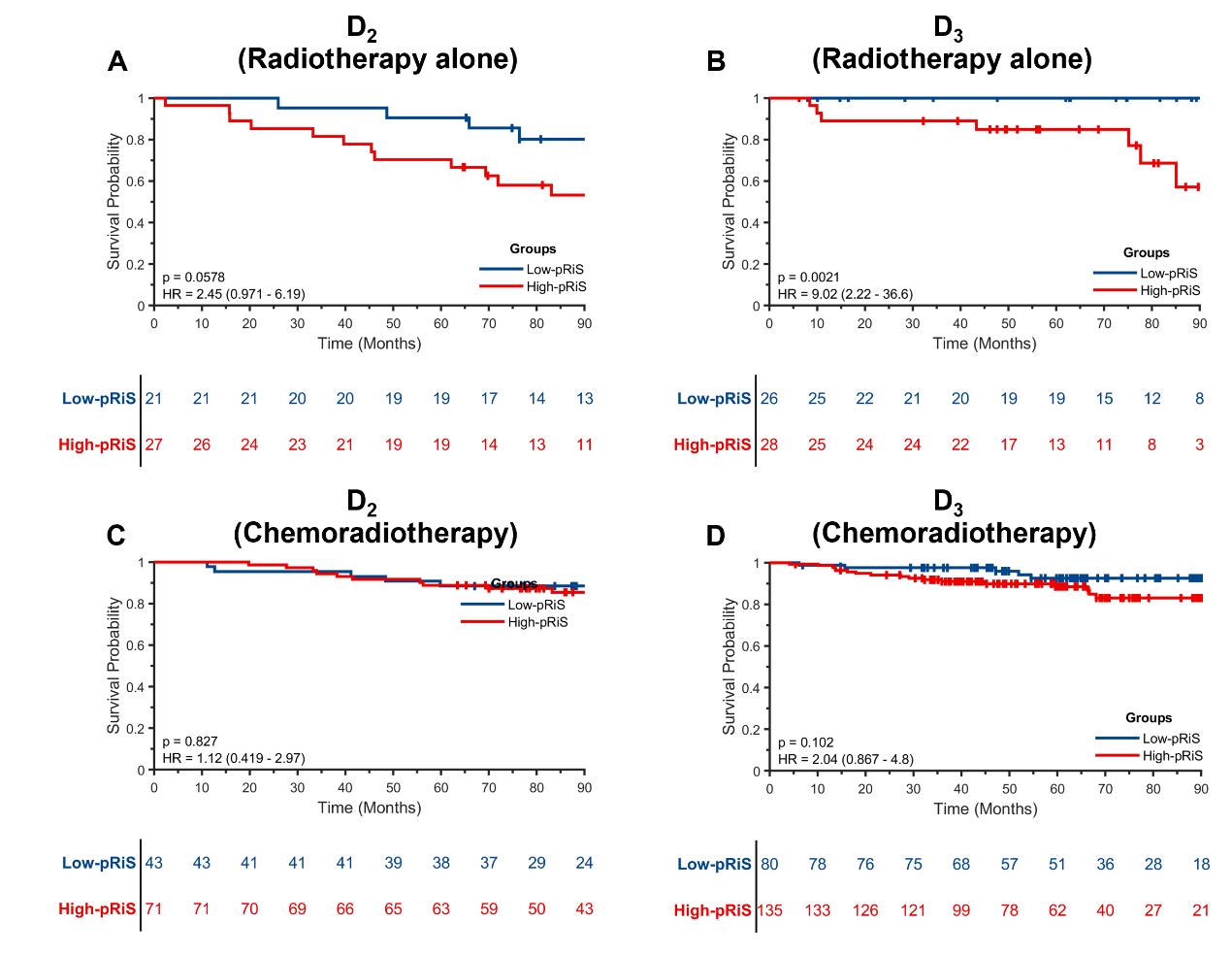


**Supplementary Figure 10. Stratified Kaplan-Meier survival analysis (X-tile cutoff = -1.1) according to treatment arms using OS as endpoint on D2 and D3.** On both D2 and D3, the high-pRiS groups have significant worse OS than the low-pRiS group when treated with radiotherapy alone while the separations in the chemoradiation arm were not significant, indicating high-pRiS patients potentially benefitted from chemotherapy.


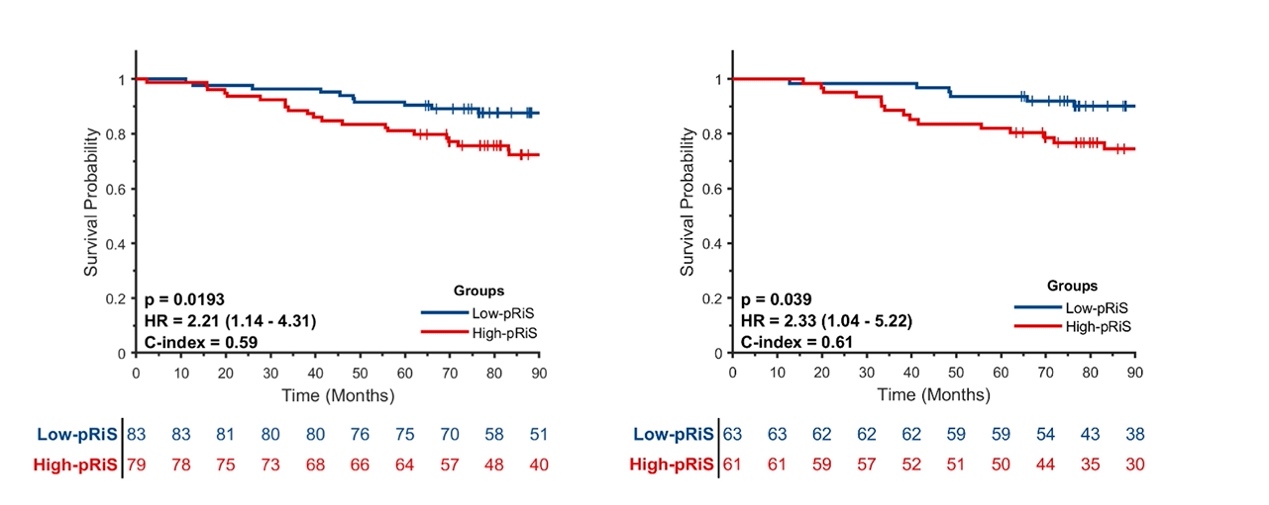


**Supplementary Figure 11. Kaplan-Meier survival analysis on D_2_ accounting for contrast injection.** After using ComBat feature harmonization (left) or eliminating scans without contrast injection (right), the stratifications on D_2_ were still significant.

Supplementary Table 1. CT imaging parameters for the three TCIA cohorts. Continuous variables are represented by mean values and ranges by (q25–q75), with q25 and q75 being the 25th and 75th percentiles, respectively.

| **Parameters** | **TCIA-OPC** | **TCIA-HNSCC** | **TICA-PET-CT** |
| --- | --- | --- | --- |
| **Image resolutions** | 0.97 (0.98–0.98) | 0.59 (0.49–0.51) | 1.06 (0.98–1.17) |
| **Slice thickness** | 2.00 (2.00–2.00) | 1.53 (1.00-2.50) | 2.89 (3.00–3.27) |
| **Manufacturer**  GE Med. Sys.  Toshiba | 151  71 | 133  1 | 44  0 |
| **Tube voltage and current** | 120 kvp  300 mAs | 120 kvp  190 ± 36 mAs | N/A |

Supplementary Table 2. Treatment details for patients included in this study.

| Cohort name | CCF  (N = 91) | TCIA-HNSCC  (n = 134) | TCIA-OPC  (n = 222) | TCIA-PETCT  (n = 44) |
| --- | --- | --- | --- | --- |
| Mean radiation dose | 70.1 | 67.8 | 67.7 | NA |
| Mean radiation number of fractions | 35.9 | 31.7 | 34.1 | NA |
| Radiation altered fractionation  Accelerated QD  BID  QD | 30  1  60 | NA | NA | NA |
| Radiation type  3DRT  IMRT  Unclear | 11  79  1 | NA | 0  222  0 | NA |
| Concurrent Chemotherapy type  CP/5FU  Carboplatin/Taxol  Cetuximab  Cisplatin  Unclear | 16  1  11  51  12 | NA | NA | NA |

Supplementary Table 3. Clinical characteristics according to treatment groups for patients in D_2_+D_3_.

| Parameter | Radiotherapy  (n = 96) | Chemoradiation  (n = 291) | P value |
| --- | --- | --- | --- |
| **Age** | 59.07 ± 9.33 | 58.23 ± 8.34 | 0.59 |
| **Gender**  Male  Female | 80 (83.3%)  16 (16.7%) | 248 (85.2%)  43 (14.8%) | 0.78 |
| **Tumor Subsite**  Base of tongue  Tonsillar complex  Posterior wall/soft palate | 42 (43.8%)  49 (51%)  5 (5.2%) | 145 (49.8%)  140 (48.1%)  6 (2.1%) | 0.2 |
| **Smoking PY** | 15.14 ± 16.59 | 12.06 ± 22.75 | 0.62 |
| **T-stage**  T1  T2  T3 | 37 (38.5%)  39 (40.6%)  20 (20.9%) | 71 (24.4%)  135 (46.4%)  85 (29.2%) | 0.02 |
| **N-stage**  N0  N1  N2 | 21 (21.9%)  44 (45.8%)  31 (32.3%) | 31 (10.7%)  160 (55%)  100 (34.3%) | 0.02 |
| **AJCC 8^th^ Stage**  I  II | 54 (56.3%)  42 (43.7%) | 123 (42.3%)  168 (57.7%) | 0.02 |

Supplementary Table 4. Top selected features from LASSO model along with their coefficients.

|  | Feature Family | Descriptor | Location | Statistics | LASSO coefficient |
| --- | --- | --- | --- | --- | --- |
| 1 | Laws | S5W5 | Intratumoral | Median | -0.0451 |
| 2 | Gabor | XY-Ang=1.1781, freq=0.276, BW=1 | 0-5 mm | Mean | 0.2316 |
| 3 | CoLlAGe | Inertia | 0-5 mm | Skewness | 0.1721 |
| 4 | CoLlAGe | Sum Average | 0-5 mm | Kurtosis | -0.7395 |
| 5 | Laws | L5R5 | 5-10 mm | Mean | 0.0016 |
| 6 | Gabor | XY-Ang=0, freq=1.276, BW=1 | 5-10 mm | Kurtosis | 0.0321 |
| 7 | CoLlAGe | Energy | 10-15 mm | Std | 8.4808 |

Supplementary Table 5. Clinical characteristics within the high- and low-pRiS groups for patients in D_1_+D_2_+D_3_.

| Parameter | high-pRiS  (n = 246) | low-pRiS  (n = 245) | P value |
| --- | --- | --- | --- |
| **Age** | 59.88 ± 8.9 | 58.16 ± 8.91 | 0.05 |
| **Gender**  Male  Female | 206 (83.7%)  40 (16.3%) | 203 (82.9%)  42 (17.1%) | 0.89 |
| **Tumor Subsite**  Base of tongue  Tonsillar complex  Posterior wall/soft palate | 118 (48%)  122 (49.6%)  6 (2.4%) | 120 (49%)  113 (46.1%)  12 (4.9%) | 0.31 |
| **Smoking PY** | 15.11 ± 20.04 | 12.02 ± 16.80 | 0.15 |
| **T-stage**  T1  T2  T3 | 63 (25.6%)  110 (44.7%)  73 (29.7%) | 80 (32.7%)  105 (42.9%)  60 (24.4%) | 0.18 |
| **N-stage**  N0  N1  N2 | 39 (15.9%)  126 (51.2%)  81 (32.9%) | 35 (14.3%)  130 (53.1%)  80 (32.6%) | 0.87 |
| **AJCC 8^th^ Stage**  I  II | 120 (48.8%)  126 (51.2%) | 121 (49.4%)  124 (50.6%) | 0.96 |
| **Recurrence**  Local/regional/distant  Non-recurrence | 33 (13.4%)  213 (86.6%) | 19 (7.8%)  226 (92.2%) | 0.06 |

Supplementary Table 6. Multivariate Cox analysis on OS in D2 and D3. Note: bold values refer to statistically significant by two-tailed test, p < 0.05.

| Parameter | D_2_ | | D_3_ | |
| --- | --- | --- | --- | --- |
|  | HR (95% CI) | P value | HR (95% CI) | P value |
| **Age** | 1.07 (1.02-1.12) | **0.007** | 0.99 (0.95-1.04) | 0.83 |
| **Gender**  Female  Male | Ref  1.16 (0.45-2.96) | 0.76 | Ref  1.89 (0.44-8.12) | 0.40 |
| **Tumor Subsite**  Base of tongue  Tonsillar complex  Posterior wall/soft palate | Ref  1.39 (0.60-3.24)  0.34 (0.04-3.06) | 0.45  0.34 | Ref  0.54 (0.23-1.27)  1.47 (0.34-1.59) | 0.16  0.99 |
| **Smoking PY** | 1.02 (1.01-1.04) | **<0.0001** | 1.02 (0.99-1.03) | 0.16 |
| **T-stage**  T1  T2  T3 | Ref  1.34 (0.41-4.40)  1.81 (0.55-5.95) | 0.32  0.1 | Ref  2.88 (0.89-9.27)  3.37 (0.87-13.01) | 0.08  0.08 |
| **N-stage**  N0  N1  N2 | Ref  1.92 (0.50-7.36)  2.85 (0.58-13.93) | 0.76  0.24 | Ref  2.9 (0.61-13.57)  1.8 (0.36-8.69) | 0.18  0.49 |
| **Treatment**  Radiotherapy  Chemoradiation | Ref  0.45 (0.21-0.96) | **0.04** | Ref  0.46 (0.17-1.22) | 0.12 |
| **pRiS** | 2.24 (1.05-4.76) | **0.04** | 7.59 (1.37-42.14) | **0.02** |

Supplementary Table 7. Point-biserial correlation coefficients between each individual prognostic radiomic feature including pRiS (rows) and the clinicopathologic factors (columns). Abbreviations: IT, intratumoral feature; PT, peritumoral feature; std, standard deviation; PY, smoking pack-year. Note: bold values refer to statistically significant values, p < 0.05.

|  | Age | PY | Gender | Tumor Subsite | Smoking History | T-stage | N-stage | AJCC 8^th^ stage |
| --- | --- | --- | --- | --- | --- | --- | --- | --- |
| IT, mean-Laws | -0.07 | **-0.12** | 0.002 | 0.08 | 0.03 | 0.07 | -0.09 | -0.001 |
| PT 0-5mm, mean-Gabor | 0.1 | **0.17** | 0.01 | 0.007 | 0.08 | **-0.12** | 0.03 | 0.01 |
| PT 0-5mm, skewness-CoLlAGe | 0.09 | -0.005 | -0.04 | **0.22** | 0.03 | 0.02 | 0.007 | **-0.09** |
| PT 0-5mm, kurtosis- CoLlAGe | **-0.12** | -0.08 | 0.04 | **0.16** | -0.02 | 0.09 | -0.07 | -0.04 |
| PT 5-10mm, mean-Laws | 0.05 | 0.05 | 0 | **-0.1** | 0.02 | -0.03 | -0.01 | -0.01 |
| PT 5-10mm, kurtosis- Gabor | -0.005 | -0.02 | 0.04 | -0.03 | 0.04 | -0.21 | 0.03 | **-0.15** |
| PT 10-15mm, std- CoLlAGe | 0.05 | 0.04 | 0.03 | -0.09 | 0.11 | -0.05 | -0.002 | -0.02 |
| pRiS | **0.17** | **0.16** | -0.003 | **-0.14** | 0.02 | **-0.2** | 0.08 | -0.03 |

Supplementary Table 8. Univariate Cox analysis on OS in D1. Abbreviations: OS, Overall survival; HR, Hazard Ratio; PY, pack-year; AJCC, American Joint Commission on Cancer; CI, confidence interval. Note: bold values refer to statistically significant by two-tailed test, p < 0.05. Significant variables were incorporated into the radiomic nomogram M_rad+c_.

| Parameter | HR (95% CI) | P value |
| --- | --- | --- |
| **Age** | 1.05 (1.01-1.09) | **0.01** |
| **Gender**  Female  Male | Ref  0.67 (0.28-1.61) | 0.37 |
| **Tumor Subsite**  Base of tongue  Tonsillar complex  Posterior wall/soft palate | Ref  0.92 (0.41-2.06)  1.47 (0.39-5.50) | 0.83  0.57 |
| **Smoking PY** | 1.03 (1.007-1.04) | **0.007** |
| **T-stage**  T1  T2  T3 | Ref  2.04 (0.66-6.26)  4.03 (1.24-13.07) | 0.22  **0.02** |
| **N-stage**  N0  N1  N2 | Ref  0.88 (0.38-2.01)  1.94 (0.64-5.87) | 0.76  0.24 |
| **AJCC 8^th^ Stage**  I  II | Ref  2.79 (1.27-6.15) | **0.01** |
| **pRiS** | 31.16 (9.15-106.1) | **<0.0001** |

Supplementary Table 9. C-indices of pRiS by scanner on the four sites. CCF cases are all scanned using the Siemens scanner, while the TCIA-HNSCC and TCIA-Head-Neck-PET-CT are using only the General Electric (GE). For OPC-Radiomic cohort, a mix of GE and Toshiba scanners were used.

| Scanner type | OPC-Radiomic | | CCF | | TCIA-HNSCC | | TCIA-Head-Neck-PET-CT | |
| --- | --- | --- | --- | --- | --- | --- | --- | --- |
|  | OS | DFS | OS | DFS | OS | DFS | OS | DFS |
| GE Med. Sys. | 0.69 | 0.64 | - | - | 0.66 | 0.64 | 0.66 | 0.65 |
| Toshiba | 0.66 | 0.61 | - | - | - | - | - | - |
| Siemens | - | - | 0.67 | 0.59 | - | - | - | - |
